# Dual energy metabolism of the *Campylobacterota* endosymbiont in the chemosynthetic snail *Alviniconcha marisindica*

**DOI:** 10.1038/s41396-020-0605-7

**Published:** 2020-02-12

**Authors:** Junichi Miyazaki, Tetsuro Ikuta, Tomo-o Watsuji, Mariko Abe, Masahiro Yamamoto, Satoshi Nakagawa, Yoshihiro Takaki, Kentaro Nakamura, Ken Takai

**Affiliations:** 1 Super-cutting-edge Grand and Advanced Research (SUGAR) Program, Japan Agency for Marine-Earth Science & Technology (JAMSTEC), 2-15 Natsushima-cho, Yokosuka, 237-0061 Japan; 2 Marine Biodiversity and Environmental Assessment Research Center (BioEnv), Research Institute for Global Change (RIGC), Japan Agency for Marine-Earth Science & Technology (JAMSTEC), 2-15 Natsushima-cho, Yokosuka, 237-0061 Japan; 3 Laboratory of Marine Environmental Microbiology, Division of Applied Biosciences, Graduate School of Agriculture, Kyoto University, Oiwake-cho, Kitashirakawa, Sakyo-ku, Kyoto, 606-8502 Japan; 4 Department of Systems Innovation, School of Engineering, The University of Tokyo, 7-3-1 Hongo, Bunkyo-ku, Tokyo, 113-8656 Japan; 5 Present Address: Department of Food and Nutrition, Higashi-Chikushi Junior College, 5-1-1 Shimoitozu, Kitakyusyu, 803-0846 Japan

**Keywords:** Biogeochemistry, Biogeochemistry, Biogeochemistry

## Abstract

Some deep-sea chemosynthetic invertebrates and their symbiotic bacteria can use molecular hydrogen (H_2_) as their energy source. However, how much the chemosynthetic holobiont (endosymbiont-host association) physiologically depends on H_2_ oxidation has not yet been determined. Here, we demonstrate that the *Campylobacterota* endosymbionts of the gastropod *Alviniconcha marisindica* in the Kairei and Edmond fields (kAlv and eAlv populations, respectively) of the Indian Ocean, utilize H_2_ in response to their physical and environmental H_2_ conditions, although the 16S rRNA gene sequence of both the endosymbionts shared 99.6% identity. A thermodynamic calculation using in situ H_2_ and hydrogen sulfide (H_2_S) concentrations indicated that chemosynthetic symbiosis could be supported by metabolic energy via H_2_ oxidation, particularly for the kAlv holobiont. Metabolic activity measurements showed that both the living individuals and the gill tissues consumed H_2_ and H_2_S at similar levels. Moreover, a combination of fluorescence in situ hybridization, quantitative transcript analyses, and enzymatic activity measurements showed that the kAlv endosymbiont expressed the genes and enzymes for both H_2_- and sulfur-oxidations. These results suggest that both H_2_ and H_2_S could serve as the primary energy sources for the kAlv holobiont. The eAlv holobiont had the ability to utilize H_2_, but the gene expression and enzyme activity for hydrogenases were much lower than for sulfur-oxidation enzymes. These results suggest that the energy acquisitions of *A. marisindica* holobionts are dependent on H_2_- and sulfur-oxidation in the H_2_-enriched Kairei field and that the mechanism of dual metabolism is controlled by the in situ H_2_ concentration.

## Introduction

It has been about 40 years since the discovery of deep-sea chemosynthetic animals in a hydrothermal system of the Galapagos rift [1–5]. To date, more than a thousand sites, including hydrothermal vent fields, methane-seep fields, and whale fall sites, have been discovered in the global ocean and their associated chemosynthetic invertebrate communities have been identified [6]. These invertebrates harbor specific lineages of chemosynthetic bacteria in the cells of specific tissues (endosymbiosis) or on their tissues (ectosymbiosis). Most of the chemosynthetic holobionts (host-symbiont associations) are sustained by energy metabolisms of either sulfur- or methane-oxidation (single thiotrophy or methanotrophy) or both (dual thiotrophy and methanotrophy) [6]. This fact raises the question why the deep-sea chemosynthetic holobionts do not adopt H_2_ oxidation (hydrogenotrophy) as their primary energy metabolism when H_2_ represents one of the most abundant gas components of hydrothermal systems and most free-living microbial populations adopt hydrogenotrophy as their predominant energy metabolism [7–12].

Petersen et al. showed for the first time that the symbiotic deep-sea mussel, *Bathymodiolus puteoserpentis*, living in the Logatchev hydrothermal field of the Mid-Atlantic Ridge (MAR) that hosts serpentinization-driven H_2_-abundant fluids, had the ability to utilize H_2_ as its chemosynthetic energy source [13]. It was also demonstrated by fluorescent in situ hybridization (FISH) and immunohistochemistry that the sulfur-oxidizing endosymbiont cells in the gills expressed genes for H_2_ oxidation [13]. Although H_2_ oxidization was likely an advantageous energy metabolism for chemosynthetic symbiosis in the H_2_-enriched Logatchev hydrothermal environment, different *B. puteoserpentis* ecotypes and their symbionts living in other MAR H_2_-depleted hydrothermal systems also seemed to have hydrogenotrophic ability [13]. Beinart et al. extensively surveyed *Alviniconcha* spp. holobionts in the Eastern Lau Spreading Center (ELSC) by quantitative molecular approach coupled with geochemical analyses using both gastight fluid sampler and in situ electrochemical instrument [14]. They demonstrated that both the host and symbiont species were genetically distinct in accordance with the geochemical conditions of their habitats, suggesting that the biogeographical distribution of holobionts was determined by the hydrothermal fluid chemistry [14]. Subsequently, based on the newly developed in situ RNA extracting instrument and metatranscriptomic analysis, Sanders et al. also demonstrated that some of the *Sulfurimonas* endosymbionts in *Campylobacterota* [15] expressed genes of Ni–Fe-hydrogenase in the northern ELSC vent fields with relatively abundant H_2_, implying that the specific *Alviniconcha* holobionts would also utilize H_2_ as their chemosynthetic energy source [14, 16]. Ikuta et al. [17] also demonstrated that *hupL* and *hupS* genes, which encode small and large subunits of hydrogenase, were expressed in the symbiont of *Bathymodiolus septemdierum* from the Myojin Knoll hydrothermal vent site. In addition, genome-sequence analyses demonstrated that several holobionts of vent-endemic chemosynthetic invertebrates have genes that are potentially involved in H_2_-oxidization, although the functional and molecular information of these genes is still not known [18–20]. Nevertheless, no deep-sea chemosynthetic holobiont that depends only on hydrogenotrophy has yet been identified, and it is yet unresolved how much the potentially hydrogenotrophic chemosynthetic symbiosis physiologically depends on H_2_ oxidation (as compared with sulfur and/or methane oxidation) and is regulated by the physical and chemical conditions of the hydrothermal fluids and habitats.

*Alviniconcha* gastropods dwell widely in many deep-sea hydrothermal systems of the western Pacific and Indian Ocean [21–23]. Each of the *Alviniconcha* populations harbors a specific lineage of chemosynthetic endosymbiont in the gill [6, 14, 22–25], while the host *Alviniconcha* and endosymbiont species represent biogeographic diversity in different deep-sea hydrothermal systems [6, 14, 22–25]. *Alviniconcha strummeri* in the Lau Basin, *Alviniconcha hessleri* in the Mariana Trough, and *Alviniconcha kojimai* in the Manus Basin and the Lau Basin harbor predominantly gammaproteobacterial endosymbionts [23]. *Alviniconcha marisindica* in the Indian Ocean and *Alviniconcha boucheti* in the Manus Basin and the Lau Basin harbor *Campylobacterota* endosymbionts [22, 23, 26]. It has been reported that the *Campylobacterota* endosymbionts of *A. boucheti* in relatively H_2_-abundant ELSC deep-sea hydrothermal systems express the genes related with H_2_ oxidation [16].

In this study, the holobionts of *A. marisindica* in the Kairei and Edmond hydrothermal fields of Central Indian Ridge (CIR) [27, 28] are investigated. The Kairei and Edmond hydrothermal vent fields show distinct hydrothermal fluid chemistry; the endmember hydrothermal fluids in the Kairei field are highly enriched with H_2_ (3.7–8.2 mM) [29–32], while the endmember fluid in the Edmond field is relatively H_2_ depleted (22–250 μM) [29, 30, 32]. It was also demonstrated that the free-living microbial communities in the hydrothermal mixing zones of the Kairei field are mainly sustained by hydrogenotrophic chemolithoautotrophy [12, 33]. The populations of *A. marisindica* in the Kairei and Edmond hydrothermal fields (kAlv and eAlv) harbor phylogenetically related *Campylobacterota* endosymbionts [26], which are also closely related to the hydrogenotrophic and thiotrophic *Sulfurovum* sp. NBC37-1 (97.1% sequence similarity) [34]. Moreover, in *Sulfurovum* sp. NBC37-1, it was proposed that the activity of the hydrogenase, an enzyme that oxidizes H_2_, was induced and strongly elevated under the high H_2_ conditions [35].

On the basis of these observations, we hypothesized that the holobionts of *A. marisindica*, particularly kAlv, would be able to utilize H_2_ as a primary energy source. If this hypothesis would be justified, we are next interested in how both *Alviniconcha* holobionts engaged to oxidize H_2_ and reduced sulfur compounds in the H_2_-abundant and -depleted habitats. We carried out multiple analyses including onboard incubation experiments and quantitative molecular and enzymatic analyses to understand the dual energy metabolisms of the holobionts of *A. marisindica*, and to address how the configuration of dual energy metabolisms are controlled by the environmental conditions of the habitats.

## Materials and methods

### Sampling sites

**Fig. 1:**
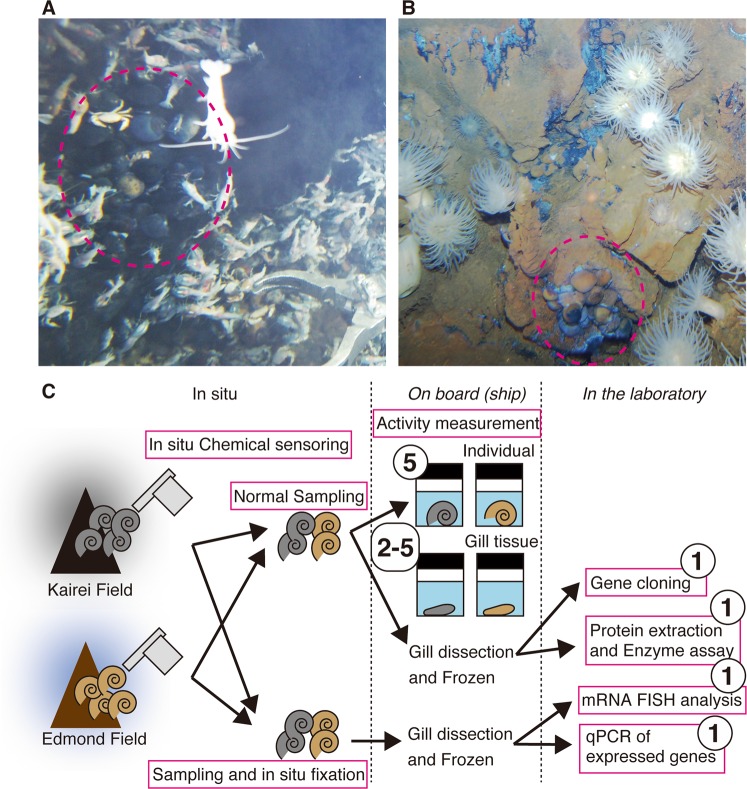
Photographs of habitats and colonies of two populations of *Alviniconcha marisindica* in the Central Indian Ridge and analytic and experimental set-ups used in this study. Magenta circles indicate the typical habitats and colonies of kAlv in the Kairei field (**a**) and of eAlv in the Edmond field (**b**). The schematic illustration shows representative experiments and analyses conducted in situ, onboard, and in the laboratory (**c**). The numbers in circles represent samples used in each experiment.

*A. marisindica* individuals and the water samples of representative habitats were collected from the Kairei (25° 19.23′ S, 70° 02.42′ E; 2415–2460 m of water depth) (Fig. 1a) and the Edmond (23° 52.68′ S, 69° 35.80′ E; 3290–3320 m of water depth) (Fig. 1b) hydrothermal vent fields in the CIR using a human-occupied vehicle, *Shinkai6500*, during the YK05-16 Leg2 (February 2006), YK09-13 Leg2 (November 2009), YK13-03 (March 2013), and YK16-E02 (February 2016) cruises of *R/V Yokosuka*. The distance between these two hydrothermal fields is 165 km. The history of this study, which explored the Kairei and Edmond fields is summarized in Table S1.

### In situ sensor measurements and water sampling

To simultaneously collect water samples surrounding the gastropod’s colonies (colony water samples) and measure the in situ H_2_ and H_2_S concentrations, we assembled a waterflow line with the in situ H_2_ and H_2_S sensor system that was equipped with a *Shinkai6500*, as summarized in Fig. S1 in Supplemental materials. Using this waterflow line, we were able to measure the chemical conditions by sensing with or without sampling water. For the in situ calibration of chemical sensors (under different temperature and pressure conditions), 5 l of chilled seawater that contained approximately 200 μM H_2_ and 500 μM Na_2_S (pH 7.5) without dissolved O_2_, or 50 μM CH_4_ with saturated dissolved O_2_, was packed in a 5-L Rontainer (Sekisui Seikei Co Ltd, Osaka, Japan), which is a thin-polyethylene container, and was deployed in the payload of *Shinkai6500* to connect the water sampling line before the dive. At the seafloor during the dive, these in situ standard waters were measured by the chemical sensors and were collected with a Hachiren water sampler. After the recovery of in situ standard water samples onboard, dissolved gas extraction from the Hachiren water samples was conducted using previously reported techniques [36] and the exact dissolved H_2_ and CH_4_ concentrations of the in situ standard water samples were determined by gas chromatography with helium ionization detector (GC-HID, GL Science, Torrance, CA, USA). The concentration of sulfide in the standard water sample was spectrophotometrically determined using the methylene blue method [37, 38]. The values of chemical sensors in the deep seawater distantly located from the deep-sea hydrothermal fields were used for the zero standard of H_2_ and H_2_S.

A phosphorescence dissolved oxygen (DO) sensor (ARO-USB, JFE Advantech Co. Ltd, Kobe, Japan) was used to measure in situ dissolved O_2_ concentration, in which the analyzed data were recorded. Measurement of DO for the invertebrate colony was carried out by directly placing this instrument on the shells of invertebrates for 5 min.

### Sampling and preparation of gastropods

Individuals of *A. marisindica* (kAlv and eAlv populations) were collected with a suction pump sampler equipped on the *Shinkai6500* from representative habitats of the Kairei and Edmond fields, respectively. As summarized in Fig. S2 of the Supplemental materials, some of the gastropod individuals were collected and then were fixed in situ by using RNA later (25 mM sodium citrate, pH 5.2, 10 mM EDTA, 0.7 kg/L ammonium sulfate) [39]. This method (without the in situ homogenization of the gastropod) did not assure appropriate fixation of the endosymbiont’s intracellular RNA by potentially incomplete infiltration of RNA later solution into the tissue, while it was confirmed that the gastropod individuals after this fixation process provided greater abundances of quantification for the 16S rRNA and other functional gene transcripts from the gill endosymbiotic cells than the individuals without the in situ fixation in the quantitative PCR (qPCR) analyses described later. After recovery onboard, most of the individuals were reared in tanks containing fresh surface seawater previously cooled at 4 °C for more than 12 h. Other individuals were dissected and the gill tissues (and some of the mantle tissues) were subsampled and stored at −80 °C and 4 °C. The frozen gill and mantle tissues were used for DNA and protein analyses.

To measure H_2_- and sulfide-consumption activity, both living *A. marisindica* individuals and dissected whole gill tissues (from individuals representing 3.5–4.0 cm shell width and 5.0–6.0 cm shell length, and within 12 h of onboard recovery) were washed twice with chilled surface seawater (previously passed through a 0.2 μm sterile filter) and incubated with substrates of either H_2_ or sulfide. In addition, the gill tissues dissected from the in situ fixed individuals were preserved into RNAprotect Bacteria Reagent (Qiagen, Valencia, CA, USA) and then stored at −80 °C for the subsequent transcript and ISH analyses. The procedure of this study is illustrated in Fig. 1c.

### Measurement of H_2_- and sulfide-consumption

Total 29 of living *Alviniconcha* individuals were acclimated in cold surface seawater at 4 °C for 12 h after recovery on the ship, then a whole individual or dissected whole gill tissue was placed in a sterile 250-ml glass bottle with 200 ml filtrated seawater and the glass bottles were sealed with a butyl rubber stopper under atmosphere. For the H_2_-consumption experiments, 5 ml of H_2_ was added to the headspace in a bottle (at a final soluble H_2_ concentration of ~100 μM) and the bottle including a living individual or gill tissue was incubated at 5 °C. At 8–12 h intervals, 1 ml of seawater was extracted from the bottle and was then injected into a 20 ml vacuumed vial. After the addition of 30 ml of helium (He) gas into the vial, 400 μl of gas phase was measured using a GC-HID. At the same time, we prepared glass bottles without individuals or gill tissue and measured the background consumption as the controls. The initial velocity of H_2_ decrease was calculated from differences between the bottles with and without *Alviniconcha* individuals. The total experiment time was set to be 40 h.

Sulfide-consumption experiments were carried out according to a method described previously [40] with minor modifications. Four milliliters of neutralized Na_2_S solution (10 mM) was added to a bottle (at a final sulfide concentration of ~200 μM) and the bottle, including a living individual or a gill tissue, was incubated at 5 °C. At 1-h intervals, 0.5 ml of seawater was extracted from the bottle and the sulfide concentration was measured by methylene blue reduction at an absorbance of 668 nm [37, 38]. To calculate activity, we used control bottles, which contained neither individuals nor gill tissue. This control bottle contained the same concentration of Na_2_S as the bottle including a living individual or gill tissue. Total experiment time was set to be 4 h. The initial sulfide concentration in this experiment was designed based on the in situ H_2_S concentrations determined by the sensor.

### Cloning and phylogenetic analysis of 16S rRNA, H_2_- and sulfur-oxidizing enzyme genes of gill endosymbionts and cytochrome *c* oxidoreductase I gene of host’s mitochondria

DNA extraction from 0.4 g frozen *A. marisindica* gill or mantle tissue was carried out using an Ultraclean Soil DNA Isolation Kit (MO BIO Laboratories, Solana Beach, CA, USA). The 16S rRNA, H_2_-uptaking hydrogenases (*hydAB*), sulfide-quinone-reductase (*sqr*), and sulfur-oxidizing enzyme complex subunit B and C (*soxB* and *soxC*) genes of *Campylobacterota* endosymbionts were amplified with the extracted DNA from the gill tissue by PCR using LA Taq with GC buffer (Takara Bio, Otsu, Japan). In these PCRs, oligonucleotide primers were used from previously described degenerated primers (for the amplification of 16S rRNA gene [41] and *sqr* gene [42]) or were designed based on our whole-genome sequencing results (for the amplification of *hydA1B1, hydA2B2, soxB* and *C* genes, *gyrB* and *gap*; unpublished data). The cytochrome *c* oxidoreductase I (*mtCOI*) gene from host-mitochondrial DNA was also amplified using the DNA extracted from mantle tissue by PCR with EX *taq* DNA polymerase (Takara Bio). All the oligonucleotide primers and PCR conditions used in this study are summarized in Tables S2 and S3. The amplicons were purified via agarose gel electrophoresis and a MiniElute Gel Extraction Kit (Qiagen) and ligated into the pCR2.1 vector in a TA cloning kit (Invitrogen, Carlsbad, CA, USA). A clone library of the amplicon of each gene was constructed using *Escherichia coli* INVαF′ (Invitrogen) with the ligation mixture. Eight of cloned fragments for each gene were directly sequenced using Sanger et al.’s method by using Applied Biosystem 3730xl Genetic Analyzer (Thermo Fisher Scientific, Waltham, MA, USA) [43]. After confirmation by direct sequencing, the plasmids containing the correct amplicons were extracted and used for the subsequent full-length sequence and phylogenetic analyses. The determined 16S rRNA gene sequence was analyzed using the ARB software package [44]. The tree of 16S rRNA gene was constructed using the neighbor-joining method of the ARB software package. Phylogenetic analyses of *hydB*, *sqr*, *soxB*, and *mtCOI* genes were performed using the neighbor-joining method in the MEGA5 software package [45].

### RNA extraction and quantification of 16S rRNA and transcripts of *hydAB*s*, sqr*, and *soxBC* genes

RNA extraction and the subsequent reverse transcription (RT) were carried out according to the method previously described [46] with some minor modifications. RNA extracts from frozen gill tissues (0.233 g for kAlv and 1.297 g for eAlv) were subjected to in situ fixation using an RNA PowerSoil Total RNA Isolation Kit (MO BIO Laboratories). The extracted RNA (500 ng) was treated with 1 μl of 100 U/ml DNaseI (Invitrogen) overnight at room temperature. Then, RT was carried out to synthesize cDNA using a Superscript III (Invitrogen) with random hexamers according to the kit manual. The synthesized cDNA assemblage was subjected to PCR to amplify 16S rRNA*, hydAB*s*, sqr soxB*, *soxC*, *gyrB*, and *gap* genes. The primers and amplification conditions of RT-PCR are summarized in Tables S2 and S3. The amplified fragments were confirmed by cloning into the PCR2.1 vector and then sequenced according to the method described for cloning of the functional genes.

All of the qPCR analyses against the synthesized cDNA assemblages were performed with a 7500 Real Time PCR system and StepOnePlus Real Time PCR system (Applied Biosystems, Foster City, CA, USA). The primer sets and conditions of the quantitative RT-PCR are listed in Tables S2 and S3, respectively. In addition, genes encoding DNA gyrase subunit B (*gyrB*) and glyceraldehyde 3-phosphate dehydrogenase (*gap*) were used in qPCR as internal controls, which were cloned from the endosymbionts by the method as described in cloning of functional genes. Standard curves were established by plasmids described in cloning of the functional genes (Fig. S8 in the Supplement materials). These standard curves spanned eight orders of magnitude and were designed to allow the addition of 10^0^–10^−7^ μg/ml of the constructed plasmid as 1 μl of template in each PCR. Ten-microliter reaction was used with a final concentration of 1× TB Green premix EX taq, 1× ROX (Takara Bio). The PCR cycling conditions were summarized in Table S3 in the Supplemental materials. To confirm whether target and/or nontarget fragments were amplified, dissociation program was added to final steps of every PCR programs and electrophoresis was carried out against the amplicons after qPCR. Data were analyzed by the system software of the instruments and then were converted to gene copies. The coefficients of determination (*R*^*2*^) were 0.9765–0.9991.

### FISH analyses of 16S rRNA and transcripts of *hydAB* and *soxB*

RNA probes for FISH were prepared as described previously [47, 48]. To synthesize RNA probes, amplifications of 16S rRNA, *hydAB*s, and *soxB* genes were conducted by PCR using primers, which were designed based on the sequence data from Tables S2 to S3 and using the constructed plasmids as templates; then the amplicons were ligated into a pTA2 vector (Toyobo, Osaka Japan). Cloned genes were sequenced by direct sequencing of the fragments [43]. Using 200 ng of the DNA fragments as templates, which were amplified from the plasmids containing each of the gene fragments, antisense and sense RNA probes were synthesized with T3 and T7 RNA polymerase, respectively, using digoxigenin (DIG) or fluorescein RNA labeling mix (F. Hoffmann-La Roche Ltd, Basel, Switzerland).

In situ hybridization was carried out as described previously [47, 48], with the following modifications. The gill tissues excised from the in situ fixed individuals were re-fixed in Davidson’s solution (10% glycerol, 40% formaldehyde, 30% ethanol, and 30% filtered artificial seawater) at −30 °C for 24 h and at 4 °C for 16 h, followed by stepwise dehydration in an ethanol series. The fixed gill was embedded in paraffin and sliced into 4-μm transverse sections. Hybridization in the presence of both DIG- and fluorescein-labeled probes and the following washing procedures were performed at 60 °C. After two-color fluorescence staining using a TSA plus cyanine 3/fluorescein kit (PerkinElmer, MA, USA), all sections were mounted in a Vectashield (Vector Laboratories, CA, USA) and covered with a coverslip. Images were taken using a Keyence (Osaka, Japan) BZ-9000 microscope, and micrographs were processed with Adobe Photoshop CS6 (Adobe Systems, CA, USA). All of the fluorescent micrographs were obtained using the same settings.

### Activity measurement of hydrogenase and sulfur-oxidizing enzymes in crude extracts of gill

Dissected gill tissue (1.8 g each) was homogenized in liquid nitrogen in a mortar and pestle and then suspended in 8 ml of extraction buffer containing 100 mM Tris–HCl and 1 mM dithiothreitol (pH 8.0). After addition of 1.0 g of glass beads (0.710–1.18 mm diameter) into the slurries, cells were thoroughly disrupted by vortex mixing. After centrifugation at 7500 × *g* for 30 min at 4 °C, the supernatants were recovered into glass vials and used for the enzyme assays as crude extracts of gill tissues. These procedures were carried out under a 95% N_2_ and 5% H_2_ atmosphere in an anaerobic chamber (Coy Laboratory Products, Grass Lake, MI, USA).

The specific activities of hydrogenase and Sox enzymes were determined according to the following method, previously described by Yamamoto et al. [35]. All measurements were taken in an anaerobic environment. When we measured hydrogenase activity in the crude extract, 10 μl of the crude extract was added to 1 ml of the reaction mixture (100 mM MOPS, pH 7.0, 5 mM methylene blue, 1 atm of 100% H_2_) that was preincubated at assay temperature (25, 45, or 65) for 5 min, and the reaction was monitored at the same temperature following the decrease in absorbance at 665 nm. Instead of 10 μl of the crude extract, we used 10 μl of the extraction buffer without any enzyme was added to the reaction mixture as a control. Specific activity of hydrogenase was calculated from the absorption coefficient of methylene blue, 74.0 cm^2^ μmol^−1^. One unit was defined as the amount of enzyme that reduced 1 μmol of electron acceptor per min. When we measured Sox activity in crude extract, 10 μl of the crude extract was added to 1 ml of the reaction solution (100 mM HEPES, pH 8.0, 35 μM horse heart cytochrome *c* (Sigma, St. Louis, Mo., USA), 10 mM sodium thiosulfate) that was also preincubated at assay temperature (25, 45, or 65 °C) for 5 min and the reaction was monitored at the same temperature following the decrease in absorbance at 550 nm. Instead of 10 μl of the crude extract, we used 10 μl of the extraction buffer without any enzyme was added to the reaction mixture as a control. Specific activity of Sox enzyme was calculated from the absorption coefficient of cytochrome *c*, 27.8 cm^2^ μmol^−1^. One unit was defined as the amount of enzyme that reduced 1 μmol of electron acceptor per min.

Measuring of sulfide:quinone reductase (Sqr) enzyme activity in crude extract was carried out by the modified method described by Wakai et al. [49]. Assay solution (250 μl) containing 50 mM BisTris, pH 7.0, 20 mM glucose, 3 mM DUQ (decylubiquinone, SIGMA) and 10 μl of the crude extract was flashed with N_2_ gas, in which one unit of glucose oxidase and ten units of catalase was added to establish anoxic conditions. Instead of 10 μl of the crude extract, we used 10 μl of the extraction buffer without any enzyme was added to the reaction mixture as a control. This prepared assay solution was preincubated at 30 °C for 5 min, then the reaction was initiated by addition of 100 μM of Na_2_S solution and monitored at 30 °C following the decrease in absorbance at 275 nm. Specific activity of Sqr enzyme was calculated from the absorption coefficient of ubiquinone, 12.5 cm^2^ μmol^−1^. One unit was defined as the amount of enzyme that reduced 1 μmol of ubiquinone per min.

Protein concentrations in the crude extracts were measured using the bicinchoninic acid method according to the manufacturer’s manual (BCA Protein Assay Reagent Kit, Pierce, Rockford, IL, USA).

### Nucleotide sequence accession numbers

All the sequences obtained in this study were deposited into DDBJ (DNA data bank of Japan) database under accession numbers AB610411–AB610419 and LC474371–LC474378.

## Results and discussion

### Phylogenetic relationship of *Alviniconcha marisindica* holobionts in the Kairei and the Edmond fields

**Fig. 2:**
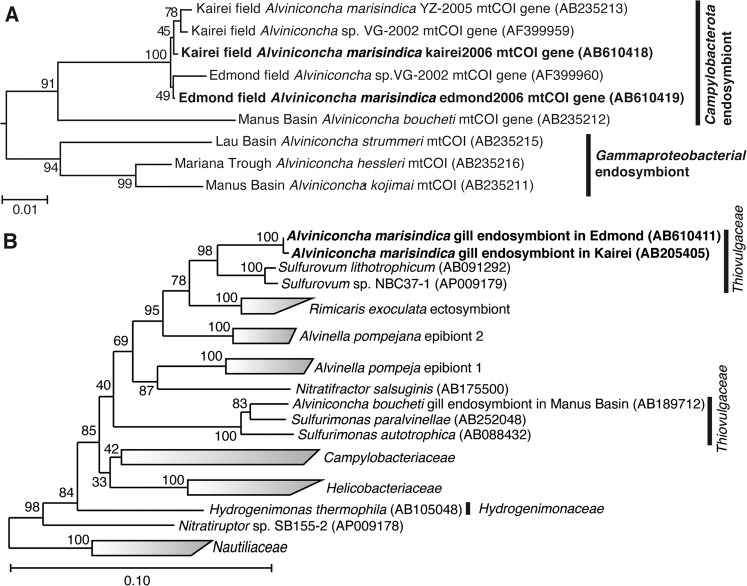
Phylogenetic trees based on the sequences of mitochondrial cytochrome *c* reductase (*mtCOI*) gene from hosts and 16S rRNA gene from endosymbionts in two populations of *Alviniconcha marisindica*. The phylogenetic trees were constructed by the neighbor-joining method using MEGA5 software [45] based on the *mtCOI* gene sequence (544 base position) (**a**) and using the ARB software package [44] based on 16S rRNA gene sequences (1402 base position) (**b**). The *mtCOI* gene sequences obtained from *A*. *marisindica* individuals in this study have been indicated in bold. The *mtCOI* genes of *Ifremeria nautilei* from North Fiji Basin (AB235214) and Manus Basin (AB235217) [22] were used as an outgroup for this tree. The 16S rRNA gene sequences obtained from *A*. *marisindica* individuals in this study have been indicated in bold. The 16S rRNA gene of *Aquifex aeolicus* (AJ309733) was used as an outgroup of this tree.

We cloned and sequenced partial mitochondrial cytochrome *c* reductase (*mtCOI*) genes from individuals of both *A. marisindica* populations. The sequence of the *mtCOI* gene of kAlv showed 99.4% sequence identity with that of eAlv, indicating that both populations of *A. marisindica* taxonomically belong to the same species. The phylogenetic tree of the *mtCOI* gene, including the data obtained from the previous studies [50], also demonstrated that kAlv and eAlv were very closely related (Fig. 2a).

The 16S rRNA genes of endosymbionts of both kAlv and eAlv were cloned and sequenced. The 16S rRNA gene sequences of the kAlv and eAlv endosymbionts determined in this study were identical to the previously reported sequences of them [26] and none of the sequences potentially derived from minor symbiontic bacteria was obtained in the previous and present studies. The sequence identity between the kAlv and eAlv endosymbionts was 99.6%, indicating that the endosymbionts of kAlv and eAlv are taxonomically the same species. The both endosymbionts belong to the genus *Sulfurovum* within the class *Campylobacterota* and the most closely related cultivated strain is *Sulfurovum* sp. NBC37-1, whose genome sequence has previously been determined [19] (Fig. 2b).

These results showed that it may be interpreted that all the molecular and functional properties of both populations in this study are derived from a taxonomically identical holobiont system, the same species of endosymbiont and host animal, although further genome-sequence analyses of both endosymbionts and hosts will clarify the genomic differences between the holobionts of kAlv and eAlv in the future.

### Physical and chemical conditions and chemolithotrophic metabolic potentials of in situ habitats of *Alviniconcha marisindica* in the Kairei and the Edmond fields

During the seafloor observation at the Kairei and Edmond hydrothermal fields, we found several *A. marisindica* colonies (Fig. 1).

In the Kairei field, the largest colony of *A. marisindica* (kAlv) was found in the diffusing fluid flow zones at the foot of the Monju chimney site and consisted of several hundred or over a thousand individuals with *Chrysomallon squamiferum* behind the large population of *Rimicaris kairei* (shrimp) (Fig. 1a) [26, 28–30, 51, 52]. When the temperature of *A. marisindica* colony water (i.e., the seawater in and around the colony of the *A. marisindica* population) was measured by a temperature probe equipped with the top of water sampler inlet during the water sampling and sensing (at the positions attaching the shells of individuals), the temperature ranged from 11.9 to 60.0 °C (average 19.3 °C) (Table 1).

**Table 1:** Physical and chemical conditions of habitats of *Alviniconcha marisindica* populations (kAlv and eAlv populations) and *Chrysomallon squamiferum* in the Kairei and Edmond hydrothermal fields.

Sampling site	Temp. range [average]	DO range [average]	H_2_ range [average]	H_2_S range [average]
	(°C)	(μM)	(μM)	(μM)
Kairei field				
Reference bottom seawater	1.7–1.8 [1.8]	207	<0.03	<0.5
*A. marisindica* (kAlv) colony at the Monju chimney	11.9–60.0 [19.3]	104–166 [144]	20.1–44.6 [34.1] in situ	137–211 [185] in situ
			11.8–20.2 [16.5] onboard	<1.5 onboard
*C. squamiferum* colony at the Monju chimney	8.4–13.5 [12.6]	133–187 [158]	8.00–8.60 [8.40] in situ	23.5–29.6 [25.1] in situ
			6.37–10.2 [8.16] onboard	<1.5 onboard
Edmond field				
Reference bottom seawater	1.8–1.9 [1.9]	208	<0.03	<0.5
*A. marisindica* (eAlv) colony at the Giant shrimp chimney	6.5–38.1 [26.8]	147–202 [184]	0.39–0.45 [0.42] in situ	109–127 [120] in situ
			0.70–4.52 [2.52] onboard	19–26 [23.3] onboard

To determine the chemical conditions within the kAlv colony, we introduced in situ electrochemical sensors for H_2_ and H_2_S concentrations, together with a DO sensor. The H_2_ concentration of kAlv colony water was found to be 20.1–40.6 μM (average 34.1 μM) (Table 1). The H_2_S concentration of water in the kAlv colony was found to be 137–211 μM (average 185 μM) (Table 1).

In the Edmond field, we discovered many small colonies (10–20 individuals) of *A. marisindica* (eAlv) along the cracks with diffusing fluid flows at the foot of the enormous hydrothermal mound (giant shrimp chimney) (Fig. 1b). The temperature of the water in several representative colonies ranged from 6.5 to 38.1 °C (average 26.8 °C) (Table 1). The H_2_ concentration in water at the eAlv habitats ranged from 0.39 to 0.45 μM (average 0.42 μM) (Table 1), indicating that eAlv inhabits water with much lower H_2_ concentrations that kAlv. In contrast, the H_2_S concentration in the eAlv habitats ranged from 109 to 127 μM (average 120 μM), and the DO concentration was found to be 147–202 μM (average 184 μM) (Table 1). In contrast to the H_2_ concentration, both the kAlv and eAlv would colonize under similar concentrations of H_2_S and DO.

To determine the H_2_ and H_2_S concentrations in both kAlv and eAlv waters, we also used the water samples recovered onboard (Table 1). However, the H_2_ and/or H_2_S concentrations in the recovered water samples were strongly reduced probably due to microbial consumption and chemical oxidation during the recovery (Table 1).

Using these physical and chemical properties of the in situ habitats and the bimodal mixing model between each of the endmember hydrothermal fluids and ambient seawater previously described [12], we calculated potential metabolic energy (J/kg water) of H_2_ oxidization with O_2_ reduction (aerobic hydrogenotrophy) and H_2_S oxidization with O_2_ reduction (aerobic thiotrophy) for free living and/or symbiotic microbial populations in the kAlv and eAlv colonies (Table 2). The calculated energy yield of aerobic hydrogenotrophy (4.4–9.4 J/kg water) using the in situ data of the kAlv colony was considerably lower than the yield based on the mixing model calculation of the kAlv colony (24.1–41.1 J/kg water) (Table 2). In contrast, the energy yield of aerobic thiotrophy in the kAlv colony was similar between the calculations based on the in situ data (39.2–65.0 J/kg water) and the mixing model (31.8–37.9 J/kg water) (Table 2). This was because the measured in situ H_2_ concentrations were lower than the H_2_ concentrations simply expected from the bimodal mixing between the endmember fluid and the seawater. Since the diffusing fluids (even high-temperature fluids) had already experienced subseafloor-microbial activities and physical–chemical processes prior to the seafloor discharge [33, 53], the H_2_ concentrations of the seafloor diffusing fluids were strongly reduced from the initial concentrations during the seafloor mixing process. Even under the in situ H_2_ concentrations, however, aerobic hydrogenotrophy is thermodynamically energy yielding and is a competitive metabolism with aerobic thiotrophy for the free living and symbiotic microbes in the kAlv population (Table 2).

**Table 2:** Potential metabolic energy yield estimated from the measured in situ physical and chemical data and the bimodal mixing model between each of the endmember hydrothermal fluids and ambient seawater.

	Calculated temperature range (°C)	H_2_-trophy colony water (J/kg)	H_2_S-trophy colony water (J/kg)
Kairei field			
* A. marisindica* (kAlv) colony			
Using the measured in situ data	11.9–60.0	4.4–9.4	39.2–65.0
Using the bimodal mixing model	12–60	24.1–41.1	31.8–37.9
* C. squamiferum* colony			
Using the measured in situ data	8.4–13.5	1.8–2.0	18.2–23.1
Using the bimodal mixing model	8–13	14.3–26.4	37.7–38.4
Edmond field			
* A. marisindica* (eAlv) colony			
Using the measured in situ data	6.5–38.1	0.08–0.1	56.1–79.5
Using the bimodal mixing model	6–40	0.3–2.5	33.7–36.2

Similarly, the calculated energy yield of aerobic hydrogenotrophy (0.08–0.1 J/kg water) using the in situ data of the eAlv colonies was found to be considerably lower than the yield (0.3–2.5 J/kg water) based on the mixing model calculation of the eAlv colony, while the energy yield of aerobic thiotrophy was similar between the calculations based on the in situ data (56.1–79.5 J/kg water) and the mixing model (33.7–36.2 J/kg water) (Table 2). The available energy potentials of aerobic hydrogenotrophy in the eAlv colonies were much lower than those in the kAlv colony. Thus, aerobic hydrogenotrophy would be highly disadvantageous for the microbial populations of eAlv colonies, including eAlv endosymbionts, compared with aerobic thiotrophy from the thermodynamic aspect. These thermodynamic estimations suggest that hydrogenotrophic energy metabolism may be the viable option for the kAlv endosymbiont but not eAlv endosymbiont, together with the thiotrophic metabolism.

### H_2_- and sulfide-consumption experiments

Onboard H_2_- and sulfide-consumption experiments were conducted using both living *A. marisindica* individuals and dissected whole gill tissues within 12 h of onboard recovery. During the incubation of living kAlv individuals with a supply of H_2_, the dissolved H_2_ concentration constantly decreased and the average H_2_-consumption rate was calculated to be 15.7 ± 8.23 μmol/h/individual (Table 3). In the case of incubations with dissected kAlv gill tissues, the H_2_-consuming rate was evident but lower than for the living individuals (the average rate was 0.59 ± 0.45 μmol/h/gill) (Table 3). These results clearly indicate that the holobiont of kAlv consumes H_2_ in the habitats. On the other hand, during the incubations with a supply of sulfide, the average sulfide-consuming rates of kAlv individuals and dissected gill tissues were 20.3 ± 6.4 μmol/h/individual and 2.78 ± 0.92 μmol/h/gill, respectively (Table 3). These rates were slightly higher than the H_2_-consuming rates.

**Table 3:** H_2_ and sulfide-consuming rates of living individuals and dissected gill tissues of *Alviniconcha marisindica* populations.

Specimen	H_2_ consumption	H_2_S consumption
*kAlv*		
Living individual	(*n* = 3)	(*n* = 3)
(μmol/h/individual)	15.7 ± 8.23	20.3 ± 6.4
(μmol/h/g of individual)	0.44 ± 0.23	0.56 ± 0.18
Gill tissue	(*n* = 5)	(*n* = 5)
(μmol/h/gill)	0.59 ± 0.45	2.78 ± 0.92
(μmol/h/g of gill tissue)	0.11 ± 0.086	0.53 ± 0.18
*eAlv*		
Living individual	(*n* = 3)	(*n* = 3)
(μmol/h/individual)	6.0 ± 1.6	9.8 ± 0.35
(μmol/h/g of individual)	0.16 ± 0.042	0.26 ± 0.0093
Gill tissue	(*n* = 5)	(*n* = 2)
(μmol/h/gill)	0.11 ± 0.21	2.1 ± 0.47
(μmol/h/g of gill tissue)	0.024 ± 0.046	0.46 ± 0.10

The numbers in parentheses show the specimen numbers of the living individuals or gill tissues used for independent incubations.

When the living individuals and dissected gill tissues of eAlv were incubated with H_2_ and sulfide supplies, the average H_2_- and sulfide-consuming rates were found to be 6.0 ± 1.6 μmol/h/individual for H_2_ and 9.8 ± 0.35 μmol/h/individual for sulfide and 0.11 ± 0.21 μmol/h/gill for H_2_ and 2.1 ± 0.47 μmol/h/gill for sulfide (Table 3). The eAlv individuals showed one-third of the H_2_-consuming and a half of the sulfide-consuming rates of the kAlv individuals, although the dissected gill tissues of both populations showed similar H_2_- and sulfide-consuming rates (Table 3). It should be noted that experimental H_2_ concentration in the eAlv individuals and gill tissue (~100 μM) was much higher than in the natural habitat (0.4 μM, Table 1) and the experimental H_2_S concentration (~200 μM) was similar to the natural habitat (120 μM, Table 1). Thus, such a high concentration of H_2_ in the incubation would induce possible hydrogenase expression and activity of the eAlv endosymbionts during incubation, as observed in the previous study of *Campylobacterota* [35]. However, these results suggest that not only the kAlv holobiont but also the eAlv entity would have the metabolic potential to utilize both H_2_ and H_2_S in their natural habitat.

The considerably lower H_2_- and sulfide-consumption rates of the dissected gill tissues compared with the living individuals were observed in both populations of *A. marisindica*. One explanation for this may be the potentially limited supply of electron acceptors (for the most part O_2_ but possibly nitrate also) for H_2_- and sulfide-oxidation in endosymbiont cells in the dissected gill tissue due to the lack of blood ventilation. Without the blood ventilation of the living individual, electron acceptors may not be sufficiently provided to the intracellularly-immobilized bacterial cells. In addition, the lack of blood ventilation may cause a serious deficiency of endosymbiont viability and metabolic activity in the absence of certain physiological factors from the host. Although we thoroughly washed the shell and body surfaces of the living individuals prior to the experiments, we cannot exclude the inevitable contribution of H_2_- and/or sulfur-oxidizing microbial populations that adhere to the shell and body surface or in the other tissues to the H_2_- and sulfide-consumption of the living individual.

### Molecular analyses of *hydABs*, *sqr*, and *soxBC* genes of endosymbionts

It is known that H_2_ oxidation is catalyzed only by a membrane-bound [Ni–Fe]-hydrogenase in the *Campylobacterota* [10, 34]. Acquired electrons from H_2_ are transferred to the terminal electron-accepting enzymes, such as polysulfide reductase, cytochrome *c* oxidase, or denitrification enzymes, potentially via ferredoxin [35]. In the draft genome sequence of the endosymbionts of kAlv, two paralogous hydrogenase genes (*hydA1B1* and *hydA2B2*) were found (unpublished data). The identity in amino acid sequence between both hydrogenases was 49% in HydA and 47% in HydB. On the other hand, sulfur oxidation is likely catalyzed by the Sox multi-enzyme system in *Sulfurovum* sp. NBC37-1, which is taxonomically closely related to the endosymbiont of *A. marisindica* [35, 39]. The Sox multi-enzyme system performs the conversion of sulfur compounds to sulfate. Acquired electrons are transferred to cytochrome *c* oxidase or denitrification enzymes potentially through cytochrome *c* [35]. The draft genome sequence of the kAlv endosymbiont demonstrated that genes encoding Sox proteins were distributed in two gene clusters (unpublished data). One consisted of *soxC*-*cyc*-*soxY*-*soxZ* genes, and the other contained *soxX*-*soxY*-*soxZ*-*soxA*-*soxB* genes. It is also known that Sqr is involved in the conversion of sulfide to polysulfide in many of the *Campylobacterota* [35, 54]. During this conversion, electrons are received by quinone and transported to the terminal enzymes. We found a *sqr* gene encoding type IV sulfide:quinone reductase in the draft genome sequence of the kAlv endosymbiont (unpublished data). These enzymes seem to be basic components for H_2_- and sulfur-oxidizing energy metabolisms of the endosymbionts of *A. marisindica*. Therefore, to evaluate the expression level of the transcripts of these functional genes related to hydrogenotrophic and thiotrophic metabolisms, we investigated *hydA1B1*, *hydA2B2*, *soxC*, *soxB*, and *sqr* genes from both endosymbionts.

The functional genes from both the endosymbionts of kAlv and eAlv of H_2_- and sulfur-oxidizing energy metabolisms such as *hydAB*s, *sqr*, and *soxBC* genes were successfully amplified using degenerated primers [41, 42] or primers based on the genome sequence from the endosymbiont of kAlv, except for the *hydA2B2* gene. We also attempted to amplify the *hydA2B2* gene using several primers inferred from the kAlv endosymbiont genome but could not obtain the gene from eAlv endosymbiont (Fig. S3). Thus, we tentatively conclude that the eAlv endosymbiont either has nonhomologous *hydA2B2* genes or lacks the *hydA2B2* gene. The *hydA1B1*, *sqr*, *soxB*, and *soxC* gene sequences from both endosymbionts were determined. The amino acid sequence identities of *hydA1B1*, *sqr*, *soxB*, and *soxC* between the kAlv and eAlv endosymbionts were 99.8% (1 amino acid difference per 149 amino acids on HydB), 99.5% (2 amino acid differences per 247 amino acids), 99.9% (1 amino acid difference per 257 amino acids), and 100% (0 amino acid differences per 231 amino acids), respectively.

In addition to the 16S rRNA gene sequence comparison, these results reinforce the idea that both endosymbionts are equipped with almost identical gene repertoires for H_2_- and sulfur-oxidizing energy metabolisms, except for the hydrogenase paralogs. In other words, the different metabolic behaviors between the holobionts of kAlv and eAlv are derived primarily from regulation at the transcription, expression, and modification levels, responding to the environmental conditions rather than from the genome and genetic structures. Phylogenetic analysis also showed that these genes from the endosymbionts are closely related with those of *Sulfurovum* species, sharing more than 97% 16S rRNA identity with the endosymbionts of both kAlv and eAlv (Figs. S4–S7). Based on the phylogenetic tree of HydB, it was suggested that the H_2_-oxidization of *Campylobacterota* could be attributed to the products of *hydA1B1* genes (Fig. S3), and we expected that the *hydA2B2* gene products were not responsible for the H_2_-oxidization of the kAlv holobiont. However, as the functions of the *hydA2B2* gene products are unknown, we conducted analyses using both *hydA1B1* and *hydA2B2* genes.

In addition, to normalize the comparative expression abundance of the functional genes between both kAlv and eAlv endosymbionts, we used *gyrB* and *gap* genes, which encode the DNA gyrase B subunit and glyceraldehyde 3-phosphate dehydrogenase, respectively. These genes are widely used as internal controls for the functional gene expression of bacteria and humans [55–58]. The amplified fragments of *gyrB* (695 bp) and *gap* (620 bp) shared 99.6% (three-bases mismatch) and 99.8% (one-base mismatch), respectively, with the endosymbionts.

### Estimation of cellular expression of functional gene transcripts in endosymbionts

**Fig. 3:**
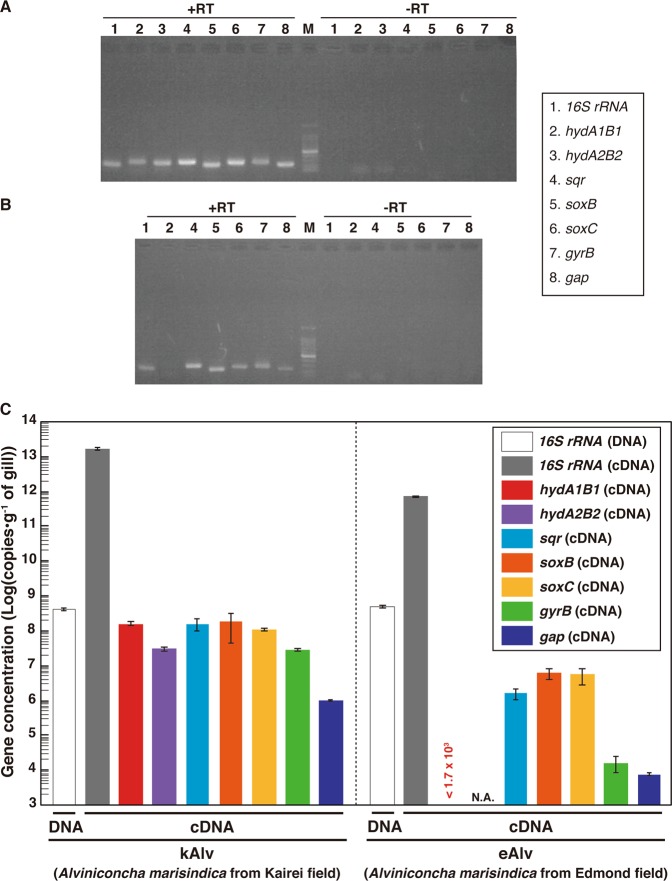
Quantitative analyses of 16S rRNA and transcripts of *hydAB*s, *sqr*, *soxB*, *soxC*, *gyrB*, and *gap* genes by RT-PCR. **a** Agarose gel electrophoresis of PCR products for 16S rRNA, *hydAB*s, *sqr*, *soxBC*, *gyrB*, and *gap* genes with or without RT of total RNA extracts from kAlv gills. **b** Agarose gel electrophoresis of PCR products for 16S rRNA, *hydA1B*1, *sqr*, *soxBC*, *gyrB*, and *gap* genes with or without RT of total RNA extracts from eAlv gills. **c** Comparative abundances of 16S rRNA gene in the DNA extracts from kAlv and eAlv gills by quantitative PCR analysis and of *hydA1B1*, *hydA2B2*, *sqr*, *soxB*, *gyrB*, and *gap* gene transcripts in the cDNA assemblages from kAlv and eAlv gills by quantitative RT-PCR analyses. Quantity of transcript (<1.7 × 10^3^ copies/g) of *hydA1B1* gene in eAlv holobiont was lower than the detection limit by qPCR analyses. NA indicates not analyzed because the *hydA2B2* gene was not detected by PCR in the eAlv endosymbiont.

To evaluate the cellular expression of the functional genes involved in the H_2_- and sulfur-oxidizing energy metabolisms, we conducted synthesis of cDNAs derived from the transcripts of 16S rRNA, *hydAB*s*, sqr*, *soxB*, *soxC* and reference genes in the endosymbionts from individuals of both kAlv and eAlv. Total RNA assemblages were extracted from the gill tissues of both populations and RT of the total RNAs were conducted. In these procedures, we confirmed that all the double-stranded DNA was completely removed before the RT step by DNaseI digestion (Fig. 3a, b). Then, RT-PCR was performed with the synthesized cDNA assemblages using the primer sets specific to each of the target genes of 16S rRNA, *hydAB, sqr*, *soxB*, and *soxC* genes. The agarose gel electrophoresis of the RT-PCR products demonstrated that the amplifications of 16S rRNA, *sqr*, and *soxB* cDNAs were observed in both the kAlv and eAlv gills while the amplification of the *hydA1B1* gene was observed only in the kAlv gill under the experimental conditions (Fig. 3a, b).

To further estimate the expression levels of functional genes, we performed qPCR analyses. We confirmed the sensitivity and confidence of quantification in our method by using the clones constructed in the cloning step (Fig. S8). First, we conducted qPCR analysis for the 16S rRNA gene of the endosymbiont using the total DNA extracts from the kAlv and eAlv gills. The estimated quantity of 16S rRNA was 4.5 × 10^8^ and 5.4 × 10^8^ copies/g for kAlv and eAlv, respectively (Fig. 3c). This result indicated that the endosymbiont cell number in 1 g of gill tissue was similar between the kAlv and eAlv individuals. However, when qPCR analysis of endosymbiont 16S rRNA was conducted using the cDNA assemblages from the kAlv and eAlv gills, it was found that the estimated number of 16S rRNA was 22 times higher in the kAlv (1.71 × 10^13^ copies/g) than in the eAlv (7.66 × 10^11^ copies/g). This result suggested that the protein synthesis activity of the kAlv endosymbiont was much higher than that of the eAlv endosymbiont. In response to the different abundances of transcript 16S rRNA, we performed qPCR analyses of the *gyrB* and *gap* gene transcripts, which were used as references for the expression levels of constitutional genes. The results confirmed that the abundances of *gyrB* and *gap* transcripts in the kAlv endosymbiont (3.11 × 10^7^ and 9.94 × 10^5^ copies/g, respectively) were 1870 times greater in *gyrB* and 130 times greater in *gap* than those in the eAlv endosymbiont (1.66 × 10^4^ and 7.73 × 10^3^ copies/g, respectively). These results indicate that the expression abundances of constitutional genes related with cellular replication, translation, and central metabolism in the endosymbionts are greater in the kAlv than in the eAlv.

In a similar manner, the RT-qPCR indicated that all functional genes analyzed in this study were more abundantly expressed in the kAlv than in the eAlv (Fig. 3c). These results strongly suggest that the kAlv endosymbiont would be functionally more active than the eAlv endosymbiont. The largest difference of expression between the kAlv and eAlv gills was found in the *hydA1B1* gene. The number of *hydA1B1* gene transcripts for the kAlv was estimated to be 1.60 × 10^8^ copies/g, while the number for eAlv was below the detection limit (<1.7 × 10^3^ copies/g) (Fig. 3c). However, the estimated abundances of *sqr*, *soxB*, and *soxC* gene transcripts in the kAlv gill were 1.61 × 10^8^, 2.00 × 10^8^, and 1.18 × 10^8^ copies/g, respectively, which were 20–100 times greater than that of the eAlv gill (1.74 × 10^6^, 6.81 × 10^6^, and 6.04 × 10^6^ copies/g for the *sqr*, *soxB*, and *soxC* gene transcripts, respectively) (Fig. 3c). In comparison with various functional genes in the endosymbionts, these results demonstrated that the functional genes for H_2_- and sulfur-oxidizing energy metabolisms were expressed at similar levels in the kAlv gill, while the functional genes for sulfur-oxidizing energy metabolism were more abundantly expressed than for H_2_ oxidation in the eAlv gill. In addition, the expression abundance ratios of hydrogenase and sulfur-oxidizing genes against the *gap* gene were 47.1 (for *hydA1B1*), 47.5 (for *sqr*), 59.0 (for *soxB*), and 34.9 (for *soxC*) in the kAlv endosymbiont, and <0.021 (*hydA1B1*), 20.0 (*sqr*), 78.0 (*soxB*), and 69.2 (*soxC*) in the eAlv endosymbiont. These values were similar between the kAlv and eAlv endosymbionts except for the expression of *hydA1B1* gene, suggesting the possible functional relevance between energy and carbon metabolisms such as glycogenesis and glycolysis.

Although the *hydA2B2* gene was only found in the kAlv endosymbiont, the estimated abundance of the transcribed gene in the gill tissue was 3.26 × 10^7^ copies/g, 4.9 times lower than that of *hydA1B1* gene (Fig. 3c). It seems likely, therefore, that the H_2_ oxidation of the endosymbiont is primarily catalyzed by products of the *hydA1B1* gene, although the function of *hydA2B2* is still uncertain.

In the kAlv endosymbiont, the results of functional gene expressions seem to be consistent with the results of the H_2_- and sulfide-consumption experiments of the living individuals. The kAlv holobiont showed higher H_2_- and sulfide-consuming activities than the eAlv holobiont (Table 3) and showed a similar level of activity for both H_2_- and sulfide-consumption (Table 3). In contrast, although the eAlv holobiont showed slightly lower H_2_-consuming activity than sulfide-consuming activity (Table 3), the expression of *hydA1B1* genes in the eAlv endosymbiont was below detection limit (<1.7 × 10^3^ copies/g gill) (Fig. 3c). As discussed above, this difference between the onboard consuming experiment and the in situ transcript analysis would be derived from the possible experimental induction of hydrogenase expression and activity by the relatively high H_2_ concentration used for the onboard consuming experiment of eAlv holobiont. It seems likely therefore that the dual energy metabolism configuration of *A. marisindica* holobionts may be regulated by the expression of related functional genes responding to the physical and environmental H_2_ conditions of their habitats.

### FISH analyses of *hydA1B1* and *soxB* gene transcripts in gill tissue

**Fig. 4:**
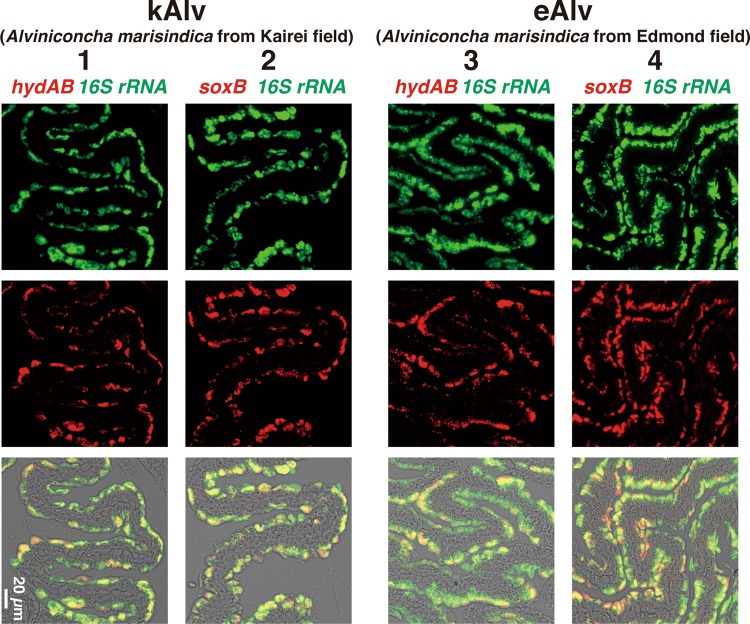
Fluorescent micrographs of gill sections of two populations of *Alviniconcha marisindica* by FISH analyses targeting 16S rRNA and functional gene transcripts. Each column shows the same section image taken by different excitation wavelengths of light. Left two columns (1 and 2) and right two columns (3 and 4) show the results of kAlv and eAlv gills, respectively. Green signals in the top panels are derived from 16S rRNA, and red signals in the middle panels are from *hydAB* gene transcripts using antisense probe (columns 1 and 3) and *soxB* gene transcripts using antisense probe (columns 2 and 4). The bottom panels show the synthesized fluorescence of 16S rRNA and each functional gene transcript.

To identify the localization of the functional gene transcripts in the gills of both populations, we designed probes to target the *hydA1B1* and *soxB* gene transcripts that were likely involved in the H_2_- and sulfur-oxidation metabolisms, respectively, and then conducted FISH analysis using the individuals fixed in situ. Consistent with the distribution pattern of endosymbionts shown in the previous study of *Alviniconcha* [59], signals for the 16S rRNA of *Campylobacterota* endosymbionts in both populations were detected throughout the apical side of the gill epithelium (Fig. 4). Similarly, signals for the *hydA1B1* and *soxB* gene transcripts were detected at the apical side of the gill epithelium in both the kAlv and eAlv, and mostly overlapped with the signals for the 16S rRNA (Fig. 4). When a sense probe was used as a control, no positive signal was observed (Fig. S9 in Supplemental materials). These results verified that both *hydA1B1* and *soxB* expression occurred not only in the kAlv endosymbiont cells but also the eAlv ones. The FISH results seem to contradict with the qPCR results indicating that the abundance of *hydA1B1* transcript in the eAlv endosymbiont was estimated as below detection limit (<1.7 × 10^3^ copies/g gill) and was much lower than that of the kAlv endosymbiont (Fig. 3c). Although other interpretations cannot be excluded, a possible explanation is that the given incubation time of catalyzed FISH in this study may have resulted in excess amplification of initial signals derived from the probes bound with the target functional gene transcripts and may not properly represent the cellular abundances of *hydA1B1* and *soxB* gene transcripts in the kAlv and eAlv endosymbionts. However, it is evident that the *hydA1B1* and *soxB* genes are co-expressed in the identical *Campylobacterota* endosymbiont cells in the *Alviniconcha* gill tissues.

### Enzymatic activity analysis in crude gill extract

To estimate the enzymatic basis of H_2_- and sulfur-oxidizing energy metabolisms in the holobionts of kAlv and eAlv, we determined specific activities of hydrogenase and Sox enzymes in the crude extracts of gill tissues from both kAlv and eAlv individuals. We also attempted to determine the specific activity of Sqr in the gill crude extracts from both populations, but it was difficult to detect the Sqr activity due to the high concentration of various types of proteins in the crude extracts, which interfered with the spectroscopic measurement of the reduced form of quinone.

**Fig. 5:**
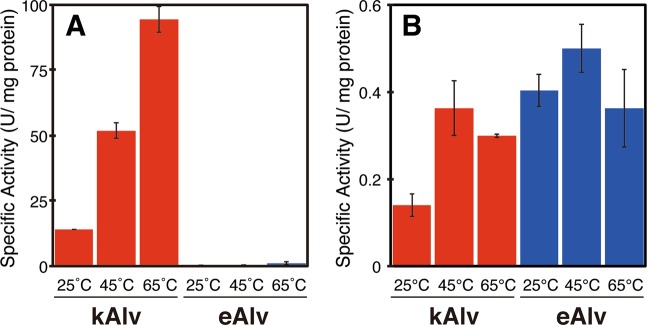
Specific activities of hydrogenase and Sox enzymes in crude extracts of gills. The activities of both enzymes were measured at 25 °C, 45 °C, and 65 °C. Red and blue bars indicate the specific activities of hydrogenase (**a**) and Sox enzymes (**b**) in crude extracts of gills from kAlv and eAlv, respectively.

The specific activity of hydrogenase in the kAlv gill crude extract was found to be 14.1, 51.8, and 94.4 U/g at 25 °C, 45 °C, and 65 °C, respectively (Fig. 5a). In contrast, the specific activity of hydrogenase in the eAlv gill crude extract was 0.025, 0.16, and 1.22 U/g at 25 °C, 45 °C and 65 °C, respectively. It was interesting that the hydrogenase activities of *A. marisindica* endosymbionts were thermophilic despite the average in situ temperatures of their habitats (20–30 °C) (Table 3). The comparison of hydrogenase activities in the gills clearly indicated that the kAlv holobiont had much greater activity (>100 times) than the eAlv holobiont. The difference of hydrogenase activity between the kAlv and eAlv holobionts was consistent with the difference in *hydA1B1* gene expression level between them (Figs. 3 and 5a). Although the living kAlv individuals or their dissected gills showed only three to six times higher H_2_-consumption activity than living eAlv individuals or their dissected gills under the H_2_-supplemented incubation condition (Table 3), the gill tissues of freshly sampled individuals from the habitats showed drastic differences in expression and activity of the H_2_-oxidizing enzyme between the kAlv and eAlv holobionts. The differences observed in H_2_-consuming experiments using individuals or gill may be due to the initial hydrogenase abundance in the endosymbiont shown in this enzyme assay using crude extract.

On the other hand, the specific activities of Sox enzymes in the kAlv gill crude extracts were 0.140, 0.362, and 0.299 U/g at 25 °C, 45 °C, and 65 °C, respectively, while those in the eAlv gill crude extract were found to be 0.404, 0.500, and 0.362 U/g at 25 °C, 45 °C, and 65 °C, respectively (Fig. 5b). The Sox enzymes from both the kAlv and the eAlv gills showed the highest specific activity at 45 °C, despite the highest activity of hydrogenase observed at 65 °C. This result may represent the substantial enzymatic property of Sox enzymes of endosymbionts, while it is probably due to an analytical artifact because the cytochrome *c* used in the Sox enzyme activity assay is obtained from bovine serum (Sigma) and thus becomes unstable at 65 °C. The comparison of Sox enzyme activities in the gills indicates that both the kAlv and the eAlv holobionts had similar activities. This result slightly contradicts the comparative results of sulfide-consumption activities of living individuals and expression abundance of *soxB* gene between the kAlv and eAlv holobionts, in which the sulfide-consumption activity of living individuals and the *soxB* gene expression were greater in the kAlv holobiont than in the eAlv holobiont (Table 3 and Fig. 3b).

Since the specific activities of hydrogenase and Sox enzymes are usually proportional to the abundance of enzymes in the extract solutions and are substantially coupled with the net energy yield, the difference seen in the enzyme activities in the gills may be derived from the different cellular expression of hydrogenase and Sox enzymes potentially responding to the environmental H_2_ concentrations in their habitats.

### Conclusive remarks and future perspectives

In this study, the chemosynthetic holobionts of two populations of *A. marisindica* in the CIR deep-sea hydrothermal fields were investigated to justify the hypothesized occurrence of dual energy metabolisms (H_2_- and sulfur-oxidation). Based on the phylogenetic analysis of representative genes from the endosymbionts and hosts, the two populations (kAlv and eAlv) had approximately identical holobiont systems that consisted of the same endosymbiotic and host species (Fig. 2a, b). The polyphasic physiological and molecular analyses clearly demonstrated that the holobionts of the two populations had metabolic potentials of both H_2_- and sulfur-oxidation (Fig. 3 and Table 3) but showed different configurations and operations of the dual energy metabolisms. The results strongly suggest that the kAlv holobiont adopts H_2_ oxidation as its primary energy metabolism as much as, or possibly more than, sulfur oxidation (Figs. 3c and 5a), while the eAlv holobiont was, for the most part, dependent on sulfur oxidation for chemosynthetic production (Figs. 3c and 5a). In the previous studies of *Alviniconcha* spp. in the ESLC [14, 16], it was suggested that the energy metabolisms of holobionts were controlled by the niche separation of different genetic and metabolic holobiont types (different couples of host and symbiont species) responding to the physical and environmental H_2_ conditions of the habitats. However, in the CIR *A. marisindica*, it is shown that the energy metabolisms of holobionts are regulated by the expression and function of dual energy metabolic genes and enzymes in the same holobiont type (the same couple of host and symbiont species). However, due to the limited opportunities for, and numbers of, dive observations, onboard experiments, and samples, statistical verification of different expression and function patterns in dual energy metabolisms was not fully conducted. In addition, as shown in the previous study of mussels in the MAR deep-sea hydrothermal fields [13], the carbon fixation and transport of holobionts associated with energy metabolisms should be clarified. These experiments will be our foci in future studies.

What would drive different configurations and operations of dual energy metabolisms in the taxonomically near-identical holobiont type of *A. marisindica*? At present, it seems very likely that the in situ H_2_ concentration of the habitat serves as one of the key factors. The kAlv holobiont dwells in habitats where the H_2_ concentration is around 100-fold higher than that of the eAlv holobiont (Table 1). If the environmental abundance of H_2_ can induce the relatively rapid change of configuration and operation of dual energy metabolisms, the hypothetical habitat exchange experiment between the kAlv and eAlv holobionts would result in the transformation of hydrogeno- and thiotrophic eAlv and thiotrophic kAlv holobionts from the native phenotypes. If other substantial factor(s) other than H_2_ (e.g., strict genotype selection and acquisition of endosymbiont at the initial holobiont formation and intergenerations of host-symbiont genetic interaction responding to the environmental conditions) can control the configuration and operation of dual energy metabolisms, the habitat exchange experiment would not affect the expression of new phenotypes. Not only habitat exchange experiments but also onboard rearing experiments under different H_2_ conditions will provide important insights into understanding the energy and carbon metabolisms of chemosynthetic symbioses in deep-sea invertebrates. For onboard rearing experiments in the future, the effect of in situ hydrostatic pressure may be considered, although it was revealed that the in situ hydrostatic pressure did not affect the specific activities of energy metabolisms in the case of the symbiotic microbial community of *Shinkaia crosnieri* (squat lobster) [39].

The H_2_-depdendent chemosynthetic symbiosis of deep-sea invertebrates was first demonstrated in a deep-sea mussel, *B. puteoserpentis*, living in the Logatchev hydrothermal field of MAR by Petersen et al. [13]. Based on the FISH and immunohistochemistry analyses and the radio-isotope-labeled bicarbonate tracer experiments, Petersen et al. [13] pointed out that hydrogenotrophy may serve as primary energy metabolism competitively with thiotrophy in the chemosynthetic holobiont. In addition, it has been reported that some chemosynthetic holobionts may use H_2_ as an energy source because the genes encoding hydrogenase, and even the expression of the genes, were found in the symbiont genomes and transcriptomes [14, 16–20]. However, it has been also shown that these holobionts have molecular and physiological potentials of other energy metabolisms (sulfur and/or methane oxidation). Actually, Mitchell et al., demonstrated that the endosymbionts of tubeworm, *Riftia pachyptila*, which possessed genes for hydrogenases did not utilized hydrogen but utilized sulfide as a major electron donor [60]. Thus, the physiological demand and metabolic response to the target energy source in the chemosynthetic holobiont should be addressed at multiple levels, including the environmental conditions of habitats, the transcription level of related genes, the expression level of related enzymes and the physiological level of individuals. In this context, we provide multiple lines of evidence that a deep-sea gastropod, *A. marisindica*, and its endosymbiont in the Kairei field utilize H_2_ as the primary energy source as well as H_2_S (sulfide).

## Supplementary information

Supplemental materials
