# Supplementary material for: Dual energy metabolism of the Campylobacterota endosymbiont in the chemosynthetic snail Alviniconcha marisindica
Source: ISME J. 2020 Feb 12;14(5):1273–89. doi: 10.1038/s41396-020-0605-7 (PMC7174374; doi:10.1038/s41396-020-0605-7)
Supplement: Supplementary file 1 — Supplemental materials [file 41396_2020_605_MOESM1_ESM.docx]

Supplemental materials for:

**Dual energy metabolisms of the *Campylobacterota* endosymbiont in the chemosynthetic snail *Alviniconcha marisindica***

Junichi Miyazaki^1^, Tetsuro Ikuta^2^, Tomo-o Watsuji^1, 5^, Mariko Abe^1^, Masahiro Yamamoto^1^, Satoshi Nakagawa^1, 3^, Yoshihiro Takaki^1^, Kentaro Nakamura^4^, Ken Takai^1^

^1^Super-cutting-edge Grand and Advanced Research (SUGAR) Program, Japan Agency for Marine-Earth Science & Technology (JAMSTEC), 2-15 Natsushima-cho, Yokosuka, 237-0061, Japan.

^2^Marine Biodiversity and Environmental Assessment Research Center (BioEnv), Research Institute for Global Change (RIGC), Japan Agency for Marine-Earth Science & Technology (JAMSTEC), 2-15 Natsushima-cho, Yokosuka, 237-0061, Japan.

^3^Laboratory of Marine Environmental Microbiology, Division of Applied Biosciences, Graduate School of Agriculture, Kyoto University, Oiwake-cho, Kitashirakawa, Sagyo-ku, Kyoto 606-8502, Japan.

^4^Department of Systems Innovation, School of Engineering, The University of Tokyo, 7-3-1 Hongo, Bunkyo-ku, Tokyo 113-8656, Japan

Corresponding author

Junichi Miyazaki

Super-Ground and -Advanced Research (SUGAR), Japan Agency for Marine-Earth Science & Technology (JAMSTEC), 2-15 Natsushima-cho, Yokosuka, 237-0061, Japan.

E-mail Address: [miyazaki11@jamstec.go.jp](mailto:miyazaki11@jamstec.go.jp)

^5^Current address

Department of Food and Nutrition, Higashi-Chikushi Junior College, 5-1-1 Shimoitozu, Kokurakita-ku, Kitakyusyu, 803-0846, Japan.

**Table S1. Expeditions and procedures in the Kairei and Edmond fields of Central Indian Ridge (CIR) for this study.**

| Month, Year | Cruise ID | The number of dives | | Conducted procedures and analyses | Remarks |
| --- | --- | --- | --- | --- | --- |
|  |  | Kairei field | Edmond field |  |  |
| February, 2006 | YK05-16 Leg2 | 5 | 5 | Water and snail sampling and gene identification |  |
| November, 2009 | YK09-13 Leg2 | 3 | 1 | On-board experiments, enzyme extraction and specific activity measurements |  |
| March, 2013 | YK13-03 | 1 | 0 | On-board experiments, and *in situ* fixation of kAlv individuals | We could not survey Edmond field due to the rough sea condition. |
| February, 2016 | YK16-E02 | 3 | 2 | Sensor measurements, sampling water and *in situ* fixation of eAlv individuals |  |

**Table S2. Primer and probe information used in this study.**

| Oligonucleotide | Sequence | Reference | Remarks |
| --- | --- | --- | --- |
| B27F | 5'-AGAGTTTGATCCTGGCTCAG-3' | 1 | Amplification of 16S rRNA gene from gill endosymbiont of *A. marisindica* |
| U1492R | 5'-ASGGNTACCTTGTTACGACTT-3' | 1 | Amplification of 16S rRNA gene from gill endosymbiont of *A. marisindica* |
| LCO1490 | 5'-GGTCAACAAATCATAAAGATATTGG-3' | 2 | Amplification of mitochondrial cytochrome c oxidoreductase I gene |
| COl-6 | 5'-GGRTARTCNSWRTANCGNCGNGGYAT-3' | 3 | Amplification of mitochondrial cytochrome c oxidoreductase I gene |
| Ge-140F | 5'-TGGATTCCRTCRAACATTTGGGT-3' | 4 | Amplification of *sqr* gene of endosymbionts |
| Ge-840R | 5'-AATWAGCATMGCRAARTCGAACTC-3' | 4 | Amplification of *sqr* gene of endosymbionts |
| EdKai_soxB200F | 5'-GATGTGATGGTGGGACACTGGGAATTT-3' | This study | Amplification of *soxB* gene of endosymbionts |
| EdKai_soxB450R | 5'-CGGTTGTACCCCATCTGTAGCCAGG-3' | This study | Amplification of *soxB* gene of endosymbionts |
| SulHynS352F | 5'-TTTTGGGACAAATACGCCTATGAAAGACCGATG-3' | This study | Amplification and quantitative PCR of *hydA1B1* gene of endosymbionts |
| SulHynL145R | 5'-ATGCAGATGGTAGAAGTGTAC-3' | This study | Amplification of *hydA1B1* gene of endosymbionts |
| AlvHydA2B2_FW3 | 5'-CGGTATAGGTTTAATGGTTGCAACGGGTG-3' | This study | Amplification and quantitative PCR of *hydA2B2* gene of endosymbionts |
| AlvHydA2B2_RV4 | 5'-CTAGGATCTCTATCTTTTGCAATTACCTC-3' | This study | Amplification and quantitative PCR of *hydA2B2* gene of endosymbionts |
| AlvHydA2B2_FW5 | 5'-GGGATACTATGGGAACAGTGAACG-3' | This study | Amplification of *hydA2B2* gene of endosymbionts |
| AlvHydA2B2_RV6 | 5'-CCAAAGCATCTTCTACTGCCATTG-3' | This study | Amplification of *hydA2B2* gene of endosymbionts |
| AlvHydA2B2_FW7 | 5'-CTTTGATAGCAGATGCCGTCAAGG-3' | This study | Amplification of *hydA2B2* gene of endosymbionts |
| AlvHydA2B2_RV8 | 5'-GCGGGAAGATTTCCATAGAGTAGG-3' | This study | Amplification of *hydA2B2* gene of endosymbionts |
| AlvSoxC_FW | 5'-AAATGGTGGACCAGAGTGGAGAAACCC-3' | This study | Amplification and quantitative PCR of *soxC* gene of endosymbionts |
| AlvSoxC_RV | 5'-GTATCCAGCATCATCTCTTGCTCTGCTG-3' | This study | Amplification of *soxC* gene of endosymbionts |
| AlvGyrB_FW | 5'-GTGGTACACATGAAGCTGGCTTTAGAG-3' | This study | Amplification and quantitative PCR of *gyrB* gene of endosymbionts |
| AlvGyrB_RV | 5'-CAATAGCGTTTGAATATGGCTCCCATC-3' | This study | Amplification of *gyrB* gene of endosymbionts |
| AlvGap_FW | 5'-GGAGTGTACGGGTGCCTTTCTTACAGC-3' | This study | Amplification and quantitative PCR of *gap* gene of endosymbionts |
| AlvGap_RV | 5'-TCACTTGAATCGTATCAAGGGGGACAA-3' | This study | Amplification of *gap* gene of endosymbionts |
| EdKai_16S_qPCR276F | 5'-TCAGCCACACTGGAACTGAG-3' | This study | Quantitative PCR of endosymbionts16S rRNA gene |
| EdKai_16S_qPCR505R | 5'-ATTCCGAGTAACGCTTGCAC-3' | This study | Quantitative PCR of endosymbionts16S rRNA gene |
| EpSqr396F_qPCR | 5'-GATTTTAGTAGGAACAGGTCACGG-3' | This study | Quantitative PCR of endosymbionts *sqr* gene |
| EpSqr688R_qPCR | 5'-CGTATTCGATTTTCCCTTCTTCTA-3' | This study | Quantitative PCR of endosymbionts *sqr* gene |
| soxB_qPCR_EdKai481F | 5'-ATCTCAAGACTCGATTTGGA-3' | This study | Quantitative PCR of endosymbionts *soxB* gene |
| soxB_qPCR_EdKai714R | 5'-ATCGCCATACCAATGAGCGA-3' | This study | Quantitative PCR of endosymbionts *soxB* gene |
| EdKai_HydS_qPCR442R | 5'-TATTTCCATTCGCGTCTGTCTTATGGAAAC-3' | This study | Quantitative PCR of endosymbionts *hydA1B1* gene |
| AlvSoxC_RV2 | 5'-GTCTGATTGGATACCCTTGCTCAGGTCTC-3' | This study | Quntitative PCR of endosymbionts *soxC* gene |
| AlvGyrB_RV2 | 5'-TTTGGCTTCTATTGGGTTTTCTTCGAGG-3' | This study | Quntitative PCR of endosymbionts *gyrB* gene |
| AlvGap_RV2 | 5'-CTTTTTTATGTCTTGCATCGAGTATAGGC-3' | This study | Quntitative PCR of endosymbionts *gap* gene |
| FISH_AlvHydAB_F | 5'-TTGGGACAAATACGCCTATGA-3' | This study | Preparation of RNA probes for *hydA1B1* gene of endosymbionts |
| FISH_AlvHydAB_R | 5'-ATGCAGATGGTAGAAGTGTACAAAAT-3' | This study | Preparation of RNA probes for *hydA1B1* gene of endosymbionts |
| FISH_AlvSoxB_F | 5'-GATGTGATGGTGGGACACTGG-3' | This study | Preparation of RNA probes for *soxB* gene of endosymbionts |
| FISH_AlvSoxB_R | 5'-CGGTTGTACCCCATCTGTAGC-3' | This study | Preparation of RNA probes for *soxB* gene of endosymbionts |
| FISH_Alv16S_F | 5'-AGCCTACCAAGCCAATGACG-3' | This study | Preparation of RNA probes for 16S rRNA gene of endosymbionts |
| FISH_Alv16S_R | 5'-AGACGAGCTCCCCAACGACT-3' | This study | Preparation of RNA probes for 16S rRNA gene of endosymbionts |

The characters S, N, R, W, Y, and M in oligonucleotide sequences indicates ambiguous bases of C/G, A/G/C/T, A/G, A/T, C/T and A/C, respectively.

**Table S3. PCR programs used in this study.**

| Target | Primers | PCR program | | | Cycling | Reference |
| --- | --- | --- | --- | --- | --- | --- |
|  |  | Denaturing step | Annealing step | Elongation Step | (Times) |  |
| *Clone analysis* |  |  |  |  |  |  |
| 16S rRNA gene of endosymbiont | B27F - U1492R | 96°C for 25 sec | 53°C for 45 sec | 72°C for 75 sec | 25 | 1 |
| mtCOI gene of host | COI-6 - LCO1490 | 94°C for 1 min | 50°C for 1 min | 72°C for 90 sec | 25 | 2 |
| *sqr* gene of endosymbiont | Ge-140F - Ge-840R | 94°C for 1 min | 45°C for 1 min | 72°C for 1 min | 50 | This study |
| *soxB* gene of endosymbiont | EdKai_soxB200F - EdKai_soxB450R | 96°C for 1 min | 50°C for 1 min | 72°C for 1 min | 50 | This study |
| *hydA1B1* gene of endosymbiont | SulHynS352F - SulHynL145R | 96°C for 1 min | 45°C for 1 min | 72°C for 1 min | 50 | This study |
| *hydA2B2* gene of endosymbiont | AlvHydA2B2_FW5 - AlvHydA2B2_RV6 | 96°C for 30 sec | 55°C for 34 sec | 72°C for 34 sec | 40 | This study |
| *hydA2* gene of endosymbiont | AlvHydA2B2_FW7 - AlvHydA2B2_RV8 | 96°C for 20 sec | 68°C for 35 sec | | 40 | This study |
| *soxC* gene of endosymbiont | AlvSoxC_FW - AlvSoxC_RV | 96°C for 30 sec | 55°C for 34 sec | 72°C for 1 min | 40 | This study |
| *gyrB* gene of endosymbiont | AlvGyrB_FW - AlvGyrB_RV | 96°C for 30 sec | 55°C for 34 sec | 72°C for 1 min | 40 | This study |
| *gap* gene of endosymbiont | AlvGap_FW - AlvGap_RV | 96°C for 30 sec | 55°C for 34 sec | 72°C for 1 min | 40 | This study |
| *RT-PCR and Quantitative PCR** | | | | | | |
| 16S rRNA gene of endosymbiont | EdKai_16S_qPCR276F - EdKai_16S_qPCR505R | 96°C for 25 sec | 55°C for 45 sec | 72°C for 34 sec | 40 | This study |
| *sqr* gene of endosymbiont | EpSqr396F_qPCR - EpSqr688R_qPCR | 95°C for 1 min | 60°C for 1 min | 72°C for 34 sec | 40 | This study |
| *soxB* gene of endosymbiont | soxB_qPCR_EdKai481F - soxB_qPCR_EdKai714R | 96°C for 30 sec | 55°C for 30 sec | 72°C for 34 sec | 40 | This study |
| *hydA1B1* gene of endosymbiont | SulHynS352F - EdKai_HydS_qPCR442R | 95°C for 30 sec | 55°C for 30 sec | 72°C for 34 sec | 40 | This study |
| *hydA2B2* gene of endosymbiont | AlvHydA2B2_FW3 - AlvHydA2B2_RV4 | 95°C for 30 sec | 55°C for 34 sec | 72°C for 34 sec | 40 | This study |
| *soxC* gene of endosymbiont | AlvSoxC_FW - AlvSoxC_RV2 | 95°C for 30 sec | 55°C for 34 sec | 72°C for 34 sec | 40 | This study |
| *gyrB* gene of endosymbiont | AlvGyrB_FW - AlvGyrB_RV2 | 95°C for 30 sec | 55°C for 34 sec | 72°C for 34 sec | 40 | This study |
| *gap* gene of endosymbiont | AlvGap_FW - AlvGap_RV2 | 95°C for 30 sec | 55°C for 34 sec | 72°C for 34 sec | 40 | This study |
| *Preparation of RNA probes* | | | | | | |
| 16S rRNA gene of endosymbiont | FISH_Alv16S_F - FISH_Alv16S_R | 96°C for 25 sec | 53°C for 45 sec | 72°C for 1 min | 40 | This study |
| *soxB* gene of endosymbiont | FISH_AlvSoxB_F - FISH_AlvSoxB_R | 96°C for 1 min | 50°C for 1 min | 72°C for 1 min | 40 | This study |
| *hydAB* gene of endosymbiont | FISH_AlvHydAB_F - FISH_AlvHydAB_R | 96°C for 1 min | 55°C for 1 min | 72°C for 1 min | 40 | This study |

*In the case of conducting quantitative PCR, the additional dissociation steps (step 1 at 95°C for 15 sec, step 2 at 60°C for 1 min, step 3 at 95°C for 15 sec) were programed after the PCR reactions.

**
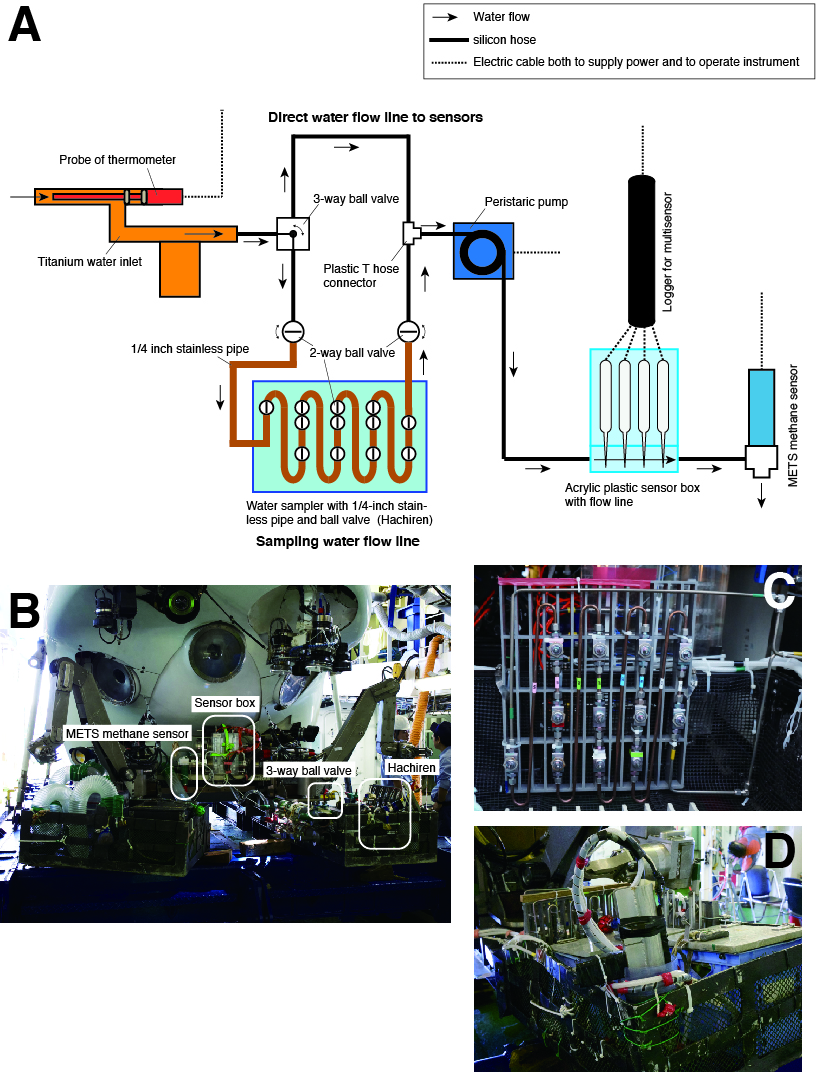
**

**Fig. S1. Waterflow line for water sampling and *in situ* sensoring equipped with *Shikai6500*.**

A. Schematic diagram of waterflow line used in this study. B. Waterflow line equipped with *Shinkai6500*. C. Hachiren water sampler [5]. D. Titanium water inlet with a thermometer probe.

In the waterflow line (Fig. S1A), the target water samples were continuously sampled by a titanium water inlet inserted with platinum-resistance electronic temperature sensor (Nichiyu-Giken, Kawagoe, Japan) [6] by the function of peristaltic pump (Nichiyu-Giken, Kawagoe, Japan). Then, by operating three-way valve, the water was introduced to the peristaltic pump either via the water sampler (*Hachiren*), which consisted of quarter-inch stainless steel pipe coated with sulfinert (GL Sciences Inc. Tokyo, Japan) and ball-valve (Swagelok, Solon, Ohio, USA) [5] or not via *Hachiren*. After passing through the peristaltic pump, a small volume of flow water (4 x 10 ml) was led to an *in situ* chemical sensor system in which two electrodes of H_2_ and H_2_S (Unisense, Aarhus, Denmark) each were set. Finally, the water was introduced to a methane sensor (METS methane sensor, Franatech, Lüneburg, Germany), which was inserted at the end of waterflow line. The H_2_ and H_2_S electrodes were electrically connected to a Field Data Logger (Unisense), in which the chemical data were recorded during the *Shinkai6500* dive. The methane sensor data were directly transferred to PC in the cockpit of *Shinkai6500* and monitored and recorded during the dive (unfortunately the methane sensor was out of order during the cruises). Water sampling and chemical sensing were carried out with a flow rate of ~150 mL/min.

**
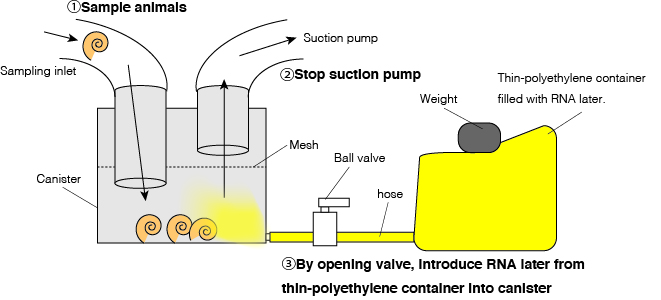
**

**Fig. S2. A schematic illustration for *in situ* RNA fixation of gastropods.**

*In situ* RNA fixation of gastropod was carried out as described in literature [7]. First, *Alviniconcha marisindica* individuals were sucked and captured in a canister (5 L volume). After stopping the suction, we introduced RNA later solution (25 mM sodium citrate, pH5.2, 10 mM EDTA, 0.7 kg/L ammonium sulfate) colored by yellow of phenol red into the canister by opening valves connecting canister to 10 L of thin-polyethylene container (Rontainer (Sekisui Seikei Co Ltd., Osaka, Japan)) filled with RNA later. In the canister, the sea water was substituted by RNA later because of a greater density of RNA later.


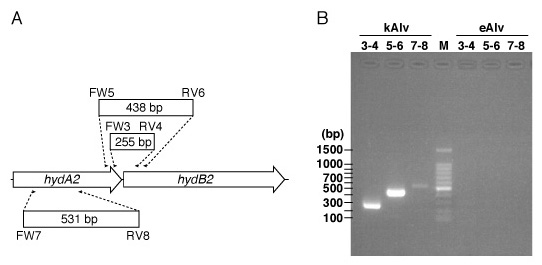
**Fig. S3. *hydA2B2* gene cloning from both *Alviniconcha* endosymbionts.**

We found two hydrogenase paralogs (named *hydA1B1* and *hydA2B2*) from on-going genomic sequence analysis of the endosymbiont of kAlv population, and therefore we tried to amplify the both genes from the DNA extracts of kAlv and eAlv gills. As illustrated in Fig. S1 (A), 3 pairs of PCR primers (Table S1) were designed and used to the PCR amplifications (Table S2). Although we successfully obtained the amplified fragments from the genome of endosymbiont of kAlv by the PCR, we could not obtain the amplicons from that of eAlv ecotype (Fig. S1 (B)). Thus, we tentatively concluded that the endosymbiont of eAlv population either has non-homologous *hydA2B2* gene to that of kAlv population endosymbiont or loses the *hydA2B2* genes in its genome. We did not conduct the subsequent analysis for the *hydA2B2* gene of eAlv population.


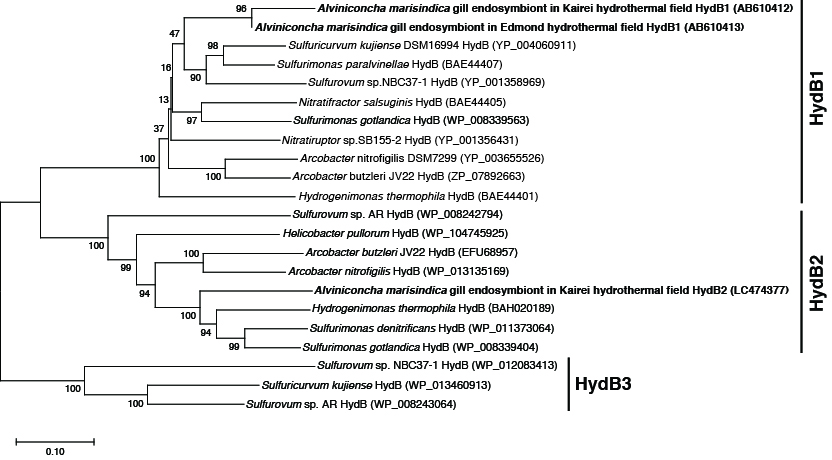


**Fig. S4. Non-rooted phylogenetic tree of deduced amino acid sequence of large subunit of hydrogenase from *Epsilonbacteraeota*.**

The tree was constructed by using software MEGA5 [8] based on deduced amino acid sequences. The HydB proteins obtained in this study are indicated by bold characters. Bootstrap analysis was performed with 1,000 resampled data sets. In this phylogenetic tree, the hydrogenase paralogs in *Epsilonbacteraeota* were clearly divided into 3 groups. The identified hydrogenase paralogs, HydB1 and HydB2, in this study (Bold characters) belonged to HydB1 and HydB2 groups, respectively. Recently published genome data demonstrated that some *Epsilonbacteraeota* bacteria have multiple hydrogenase genes while some of them are not able to utilize H_2_ as an electron donor for the growth (Fig. S2) [9-13]. The phylogenetic tree indicated that the hydrogenotrophically growing *Epsilonbacteraeota* bacteria always have the *hydB* gene of HydB1 group while non-hydrogenotrophic strains do not have the gene of HydB1 group. Especially *Sulfrovum* sp. AR has two hydrogenase paralogs belonging to HydB2 and HydB3 groups in its genome but this bacterium grows with thiosulfate as the sole electron donor [12]. These observations suggest that hydrogenase paralogs in HydB2 and HydB3 groups may be not responsible for the hydrogenotrophic energy metabolism. In this study, the eAlv holobiont showed H_2_-consuming activity and we detected only the *hydB1* gene belonging to HydB1 group. It seems likely therefore that the kAlv holobiont has two hydogenase paralogs, HydA1B1 and HydA2B2, but HydA1B1 primarily serves for the hydrogentrophic energy metabolism. The role of HydA2B2 is still uncertain.


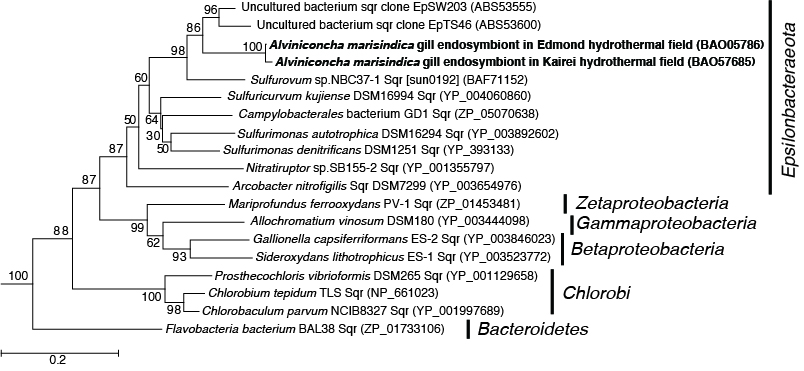


**Fig. S5. Phylogenetic tree of amino acid sequence of sulfide-quinone oxide reductase (Sqr) from various bacteria.**

The tree was constructed by using software MEGA5 [8] based on deduced amino acid sequences. The Sqr protein obtained in this study are indicated by bold characters. The amino acid sequence of conserved hypothetical protein from *Sulfurovum* sp. NBC37-1 (BAF71007) was used as the outgroup of this tree. Bootstrap analysis was performed with 1,000 resampled data sets.


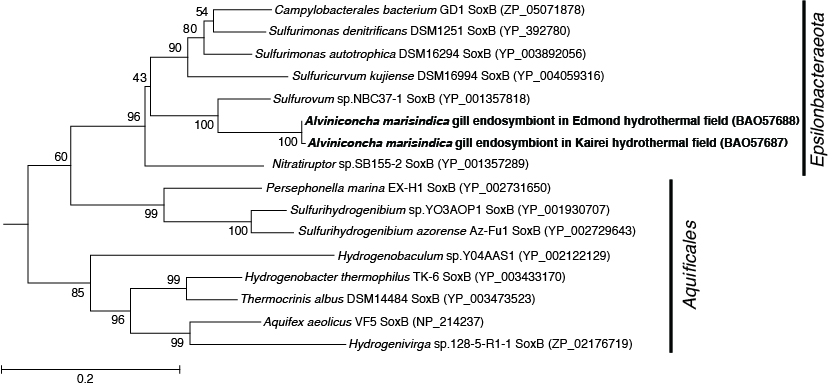


**Fig. S6. Phylogenetic tree of amino acid sequence of SoxB enzyme from *Epsilonbacteraeota* and *Aquificales*.**

The tree was constructed by using software MEGA5 [8] based on deduced amino acid sequences. The SoxB protein obtained in this study are indicated by bold characters. The amino acid sequence of chain A of 5’-nucleotidase from *Escherichia coli* (PDB:1USH) was used as the outgroup of this tree. Bootstrap analysis was performed with 1,000 resampled data sets.


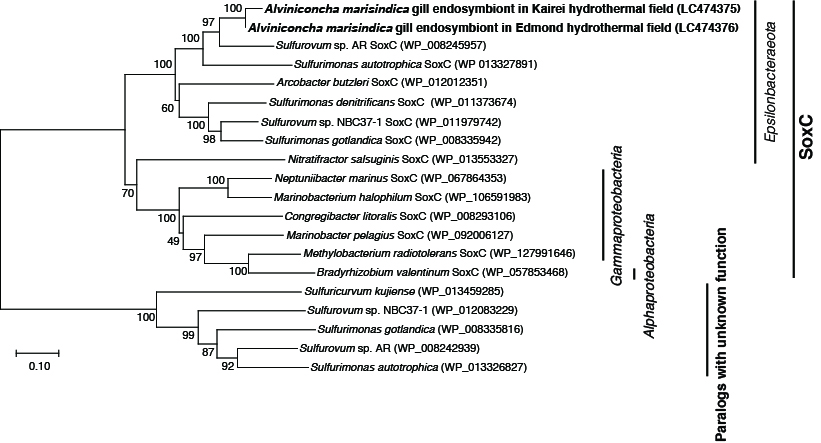


**Fig. S7. Non rooted phylogenetic tree of amino acid sequence of SoxC enzyme from *Epsilonbacteraeota* and other proteobacteria.**

The tree was constructed by using software MEGA5 [8] based on deduced amino acid sequences. The SoxC proteins obtained in this study are indicated by bold characters. We found the SoxC paralogs in the genomes of other *Epsilonbacteraeota*, while on-going genome analysis of endosymbiont in kAlv ecotype confirmed that the kAlv endosymbiont had only one *soxC* gene.


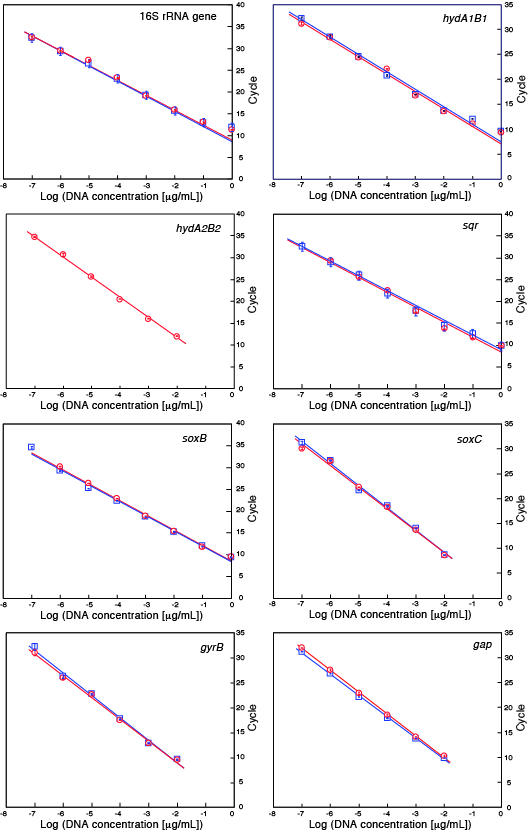


**Fig. S8. Amplification pattern by qPCRs for the cloned genes of *16S rRNA*, *hydA1B1*, *hydA2B2*, *sqr*, *soxB*, *soxC*, *gyrB*, and *gap* constructed in this study.**

These patterns were used as standards. Red lines and open circles indicate the amplification patterns of genes cloned from kAlv population, whereas blue lines and squares indicate the patterns of genes cloned from eAlv population.

**
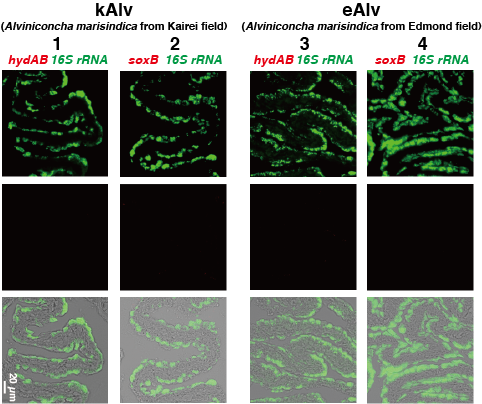
**

**Fig. S9. Negative control of fluorescent micrographs of gill sections of two populations of *Alviniconcha marisindica* by FISH analyses targeting 16S rRNA and functional gene transcripts using sense probe.**

Each column shows the same section image taken by different excitation wavelengths of light. Left two columns (1 & 2) and right two columns (3 & 4) show the results of kAlv and eAlv gills, respectively. Green signals in the top panels are derived from 16S rRNA, and red signals in the middle panels are from *hydAB* gene transcripts (columns 1 & 3) and *soxB* gene transcripts (columns 2 & 4). The bottom panels show the synthesized fluorescence of 16S rRNA and each functional gene transcript.**References**

1. DeLong EF. Archaea in coastal marine environments. *Proc Natl Acad Sci USA* 1992; 89: 5685–9.
2. Folmer O, Black M, Hoeh W, Lutz RA, Vrijenhoek RC. DNA primers for amplification of mitochondrial cyto- chrome c oxidase subunit I from diverse metazoan inverte- brates. *Mol Mar Biol Biotechnol* 1994; 3: 294–99.
3. Shimayama T, Himeno H, Sasuga J, Yokobori S, Ueda T, Watanabe K. The genetic code of a squid mitochondrial gene. *Nucleic Acids Symp Ser* 1990; 22: 77–78.
4. Pham VH, Yong JJ, Park SJ, Yoon DN, Chung WH, Rhee SK. Molecular analysis of the diversity of the sulfide:quinone reductase (*sqr*) gene in sediment environments. *Environ Microbiol* 2008; 154: 3112-21.
5. Kawagucci S, Miyazaki J, Noguchi T, Okamura K, Shibuya T, Watsuji T, *et al.* Fluid chemistry in the Solitaire and Dodo hydrothermal fields of the Central Indian Ridge. *Geofluids* 2016; 16: 988–1005.
6. Miyazaki J, Makabe A, Matsui Y, Ebina N, Tsutsumi S, Ishibashi J, *et al.* WHATS-3: An improved flow-through multi-bottle fluid sampler for deep-sea geofluid research. *Frontiers in Earth Science* 2017; 5: 202–13.
7. Watsuji T, Yamamoto A, Takaki Y, Ueda K, Kawagucci S, Takai K. Diversity and methane oxidation of active epibiotic methanotrophs on live *Shinkaia crosnieri*. *ISME J* 2014; 8: 1020-1031.
8. Tamura K, Peterson D, Peterson N, Stecher G, Nei M, Kumar S. MEGA5: Molecular evolutionary genetics analysis using maximum likelihood, evolutionary distance, and maximum parsimony methods. Molecular Biology and Evolution *Mol Biol Evol* 2011; 28: 2731-39.
9. Miller WG, Parker CT, Rubenfield M, Mendz GL, Wösten MMSM, Ussery DW, *et al*. The complete genome sequence and analysis of the *Epsilonproteobacterium* *Arcobacter butzleri*. Fairhead C, editor. *PLoS ONE.* 2007; 2: e1358–21.
10. Nakagawa S, Takaki Y, Shimamura S, Reysenbach A-L, Takai K, Horikoshi K. Deep-sea vent epsilon-proteobacterial genomes provide insights into emergence of pathogens. *Proc Natl Acad Sci USA.* 2007; 104: 12146–50.
11. Anderson I, Sikorski J, Zeytun A, Nolan M, Lapidus A, Lucas S, *et al*. Complete genome sequence of *Nitratifractor salsuginis* type strain (E9I37-1). *Stand Genomic Sci.* 2011; 4: 322–30.
12. Park S-J, Ghai R, Martin-Cuadrado A-B, Rodriguez-Valera F, Jung M-Y, Kim J-G, *et al*. Draft genome sequence of the sulfur-oxidizing bacterium “Candidatus *Sulfurovum* sediminum” AR, which belongs to the *Epsilonproteobacteria*. *J Bacteriol.* 2012; 194: 4128–9.
13. Han CS, Kotsyurbenko O, Chertkov O, Held B, Lapidus A, Nolan M, *et al*. Complete genome sequence of the sulfur compounds oxidizing chemolithoautotroph *Sulfuricurvum kujiense* type strain (YK-1(T)). *Stand Genomic Sci*. 2012; 6: 94–103.
